# Life Cycle Assessment of Lithium-Ion Battery Recycling: Evaluating the Impact of Recycling Methods and Location

**DOI:** 10.1021/acs.est.4c13838

**Published:** 2025-07-10

**Authors:** Francis Hanna, Calvin Somers, Annick Anctil

**Affiliations:** † Department of Civil & Environmental Engineering, 3078 Michigan State University, East Lansing, Michigan 48824, United States; ‡ Department of Applied Engineering, Michigan State University, East Lansing, Michigan 48824, United States

**Keywords:** Li-Ion Batteries, Recycling, Pyrometallurgy, Hydrometallurgy, Life Cycle Assessment

## Abstract

Lithium-ion battery (LIB) recycling technologies are advancing rapidly, with higher recovery efficiencies, lower energy demand, and more complex supply chains. Previous life cycle assessment (LCA) studies overlook evolving industry recycling practices and often disregard key impact categories, such as water consumption, toxicity, and resource depletion potential. Previous studies also do not evaluate battery recycling methods within the current supply chain context, specifically accounting for prevailing battery waste composition, final cathode material outputs, and varying geographic locations of recycling stages. This study compares conventional hydrometallurgy (CHR), truncated hydrometallurgy (THR), and pyrometallurgy (PR) recycling in North America, Europe, and China. This work considers each method’s recycling efficiency and the additional primary materials required to produce the new NMC811 CAM. Leaching and materials extraction contribute the most to the environmental footprint of recycling. Skipping metals’ extraction in THR leads to the lowest carbon footprint, water consumption, and toxicity. Compared to China, recycling and manufacturing in North America reduce the carbon footprint and freshwater toxicity of the NMC811 CAM by 16% and 30%, respectively. Careful selection of the recycling and production locations can reduce the environmental impact of a modern EV NMC811 battery pack by 792 kg CO_2_-eq and 11,355 L of water, respectively.

## Graphical abstract

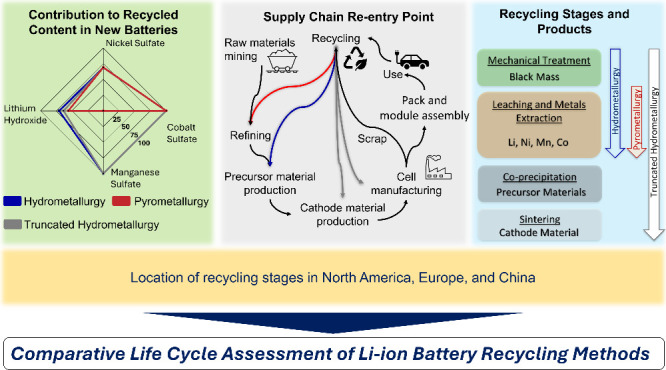

## 1 Introduction

Global demand for Li-ion batteries (LIBs) is increasing and expected to reach 4.7 TWh in 2030, primarily driven by efforts to electrify mobility and secure energy storage for renewable energy systems.^1^ These goals are vital for climate change mitigation but introduce two challenges: battery material demand and future battery waste.^2–4^ Recycling can help manage spent LIBs, offset the environmental impacts of virgin battery materials, and support a domestic material supply chain.^4,5^

Countries worldwide are updating battery regulations to retain critical resources through battery tracking and recycling.^6^ The US Inflation Reduction Act (IRA) provides investment tax credits for battery recycling projects and offers tax credits for vehicles using materials extracted or processed in the US or a free trade partner,^7,8^ encouraging recycling to reduce materials imports. Several states, including New Jersey and California, have enacted policies assigning EV manufacturers the responsibility of recycling spent LIBs.^9–11^ European Battery Regulations require EV and battery manufacturers to ensure the collection and recycling of at least 63% of batteries by 2027 and 73% by 2030,^12^ and ensure minimum recycled content of nickel, cobalt, and lithium in new batteries.^13–15^ From 2027, EU manufacturers must disclose, via a battery passport, the battery’s environmental footprint, materials’ provenance, chemical makeup, and manufacturing history.^12^

As a result, there’s a growing focus on battery recycling to provide sustainable recycled materials for the battery supply chain. Battery recycling methods fall into two broad categories: pyrometallurgy and hydrometallurgy.^16–19^ Pyrometallurgy is a mature and well-established technology that uses heat to process batteries.^18,20^ However, pyrometallurgy is energy-intensive and has a low lithium recovery efficiency. Hydrometallurgy is a highly selective recycling method that uses mechanical treatment and chemical leaching to process spent batteries.^21,22^ Conversely, hydrometallurgy is chemically intensive and produces large amounts of wastewater that must be treated or recycled. In addition to conventional recycling methods, emerging technologies aim to speed up and maximize the recovery efficiency of battery recycling and reduce its environmental impact. For example, innovative “truncated” hydrometallurgical recycling processes recover new cathode materials (CAM) using a simplified flowsheet with minimal use of intermediate battery materials, such as nickel sulfate, cobalt sulfate, or lithium carbonate.^23,24^

Life cycle assessment (LCA) is often used to investigate the environmental impact of LIB recycling.^25–36^ However, most studies do not evaluate processes that reflect the latest industry practices. For example, pyrometallurgical recycling has evolved with improved energy efficiencies and lithium recovery capabilities.^34,37,38^ The new pyrometallurgical process is autogenous via the embedded chemical energy in the batteries, potentially reducing the energy demand and carbon emissions.^34^ Previous studies do not account for these changes and potentially overestimate the environmental impacts of pyrometallurgical recycling. Numerous studies evaluate the environmental footprint of conventional hydrometallurgy recycling by retrieving their inventory from the GREET model or other public databases.^32,39,40^ But the inventories used in these studies overlook important recycling stages, such as solvent extraction, crystallization, or recovery of byproducts such as gypsum and sodium sulfate. Only one study evaluates the environmental impact of new truncated hydrometallurgical recycling and compares it with conventional hydrometallurgy.^40^ But this study compares these two recycling methods despite yielding different products. Conventional hydrometallurgy yields discrete metals, while truncated hydrometallurgy yields a soluble mix of battery materials. Here it is important to note that system boundary considerations have been another concern in previous studies.

Previous LCA studies evaluating battery recycling follow three general approaches: (1) analyze the recycling process itself,^25,34,41–44^ (2) focus on the end-use application of recycled materials,^35,45,48^ or (3) model the production and recycling of CAM and deduct the avoided burden of recycled materials.^33,45–49^ The first approach evaluates the recycling methods based on the environmental footprint of processing spent batteries, while the second approach is based on the environmental impact of producing cathode materials using 100% recycled materials content. Both approaches present important limitations. Recycling processes involve complex operations in which battery materials are recovered in various stages, each with distinct material and energy demands. Consequently, assessments that focus only on the recycling process fail to provide meaningful insights into the actual environmental footprint of the recovered materials and their potential to reduce the impact of future battery production. Conversely, assuming cathode material production with 100% recycled content is not realistic given the current industry constraints.^50^ Additionally, assuming 100% recycled content overlooks the effect of recycling efficiency, which affects the extent to which recycled materials can effectively reduce the demand for primary resources. The third approach provides little clarity on the actual environmental impacts of the battery recycling process because only the new CAM environmental impact is reported. Thus, a comprehensive assessment is needed that explicitly integrates recycling processes, CAM production, and supplemental primary material inputs. Additionally, studies must ensure that battery waste composition and output CAM chemistry assumptions reflect realistic market trends and foreseeable industry developments. By inclusion of these improvements, assessments can more accurately represent real-world conditions, offering more meaningful insights into the true environmental implications of battery recycling strategies.

Battery recycling is also evolving rapidly, with complex supply chain models. For example, the Li-cycle implements the hub-and-spoke supply chain model where spokes process spent Li-ion batteries at different locations to produce the black mass, which is sent to a central hub for further processing and metal extraction.^51^ Previous studies did not analyze the effect of such supply chain complexities on the environmental impact of recycling. Thus, a comprehensive assessment is needed to model these variations of the battery recycling and production supply chain.

The objective of this study is to provide an updated inventory and comprehensive environmental impact assessment comparing conventional hydrometallurgy, pyrometallurgy, and truncated hydrometallurgy recycling methods. These recycling techniques are evaluated across North America, Europe, and China within the context of current supply chains, specifically accounting for prevailing battery waste composition, final cathode material outputs, and geographic location of recycling stages—a key differentiating factor between conventional and emerging recycling technologies. Finally, the study analyzes existing and prospective policy frameworks aimed at reducing the environmental footprint associated with battery recycling processes and cathode active material (CAM) production.

## 2 Methods

### 2.1 Goal and Scope

This study aims to assess the impact of recycling technology and location on the environmental impact of Li-ion battery pack (LIBP) recycling. We perform an LCA of NMC LIBP recycling in the US, Europe, and China using pyrometallurgical, conventional hydrometallurgical, and truncated hydrometallurgical recycling.

### 2.2 System Boundary and Functional Unit

The system boundary includes three phases: recycling, precursor material production (pCAM), and CAM production (Figure 1). The process starts with recycling spent NMC LIBP via hydrometallurgy, pyrometallurgy, or truncated hydrometallurgy recycling. The recovered metal sulfates are used to produce NMC811 pCAM. Then the pCAM is lithiated using recycled and virgin lithium hydroxide to produce NMC811 CAM. We assume the battery waste mix includes NMC811 from manufacturing scrap (70%) and retired NMC622 batteries (30%). Following the gradual transition toward low-cobalt batteries, NMC811 is the final output in this analysis.^1,52^

**Figure 1:**
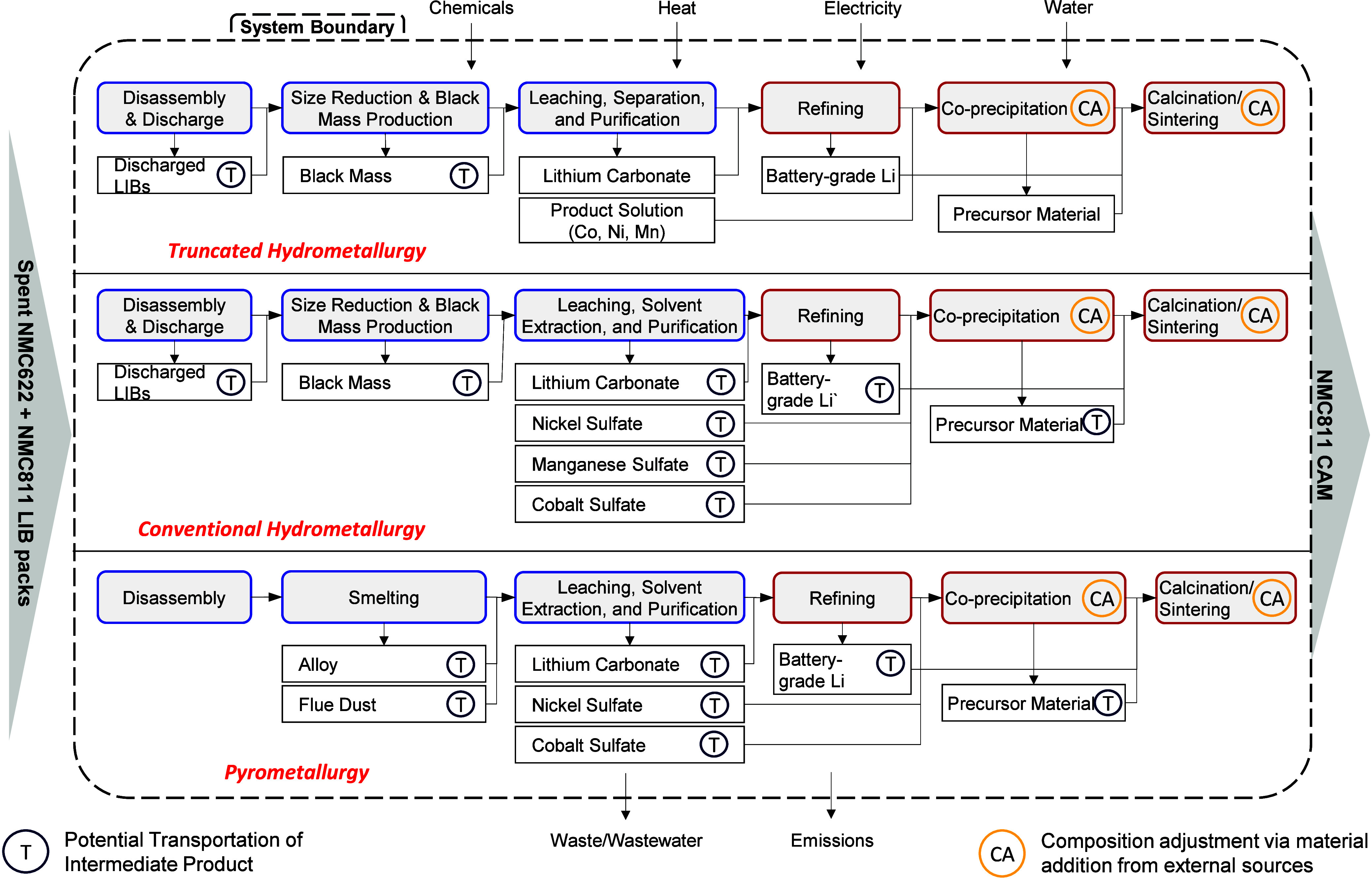
System boundary of study—grave-to-gate process for LIBP recycling and CAM production. FU1:1 kg of NMC LIBP recycled; FU2:1 kg of NMC811 CAM produced. Note: This figure does not reflect the noncathode materials flow. SI1.1 summarizes the fate and recovery assumptions of noncell materials.

The system boundary does not include the first life cycle and the collection and transportation of spent LIBs to narrow the focus of this article on battery recycling and production. The impact of the use phase depends on many factors, such as the vehicle characteristics, the driving cycles, and the source of electricity used to charge the battery.^34,53,54^ We assume virgin materials are used for composition adjustment in the second and third phases. SI-1 sections 1 and 2 summarize the virgin materials’ sources and the location assumptions of each processing stage.

This study considers two functional units (FU): 1 kg of NMC LIBP recycled and 1 kg of NMC811 CAM produced. The first F.U. allows us to assess the footprint of recycling 1 kg LIB packs and is selected to facilitate comparison with previous studies that have used the same (SI1 section 6). The second FU extends the analysis to assess the effect of the recycling method selection on the environmental footprint of future CAM. Particularly, the second FU reflects the environmental footprint of the recycled and additional primary materials needed in the new CAM.

### 2.3 LIB Recycling Methods and Scenario Development

This study analyzes three recycling methods: conventional hydrometallurgy (CHR), pyrometallurgy (PR), and truncated hydrometallurgy (THR). The assessed recycling methods are evaluated using the patents of industrial companies that commercialized the processes.^24,37,38,55,56^ SI-1 section 3 details the modeled recycling methods and their respective process diagrams.

CHR consists of three primary stages: (1) disassembly and discharge, (2) size reduction and black mass generation, and (3) leaching and solvent extraction. The final stage results in the extraction of battery-grade metal sulfates and lithium carbonate. THR also involves discharge, disassembly, size reduction, black mass generation, and leaching. In contrast with CHR, metal sulfates are not extracted and separated following leaching. Instead, they remain mixed in aqueous sulfate solution and adjusted to meet the stoichiometry requirements of the final ternary CAM, NMC811. In PR, battery packs are first disassembled to remove the casing material and then directly undergo a smelting step, producing slag, alloy, and flue dust. Cobalt and nickel sulfate are recovered from the alloy via solvent extraction and crystallization. Lithium carbonate is recovered from the flue dust via carbonated water leaching.

Recycling and production stages can happen in different locations, which is a common practice in the industry. The current study evaluates battery recycling and CAM production in North America, Europe, and China based on existing supply chain scenarios. Of note, the scenarios are not exhaustive and might not be fully representative of battery supply chains, particularly in North America and Europe. A sensitivity analysis (section 2.6) is conducted to better understand the impact of recycling method and location. SI-1 section 2 summarizes the scenario development and the respective location of each recycling and production stage. Here it is important to clarify that we use “supply chain” in the current study to refer to regional configuration of process locations and energy mixes. Additional supply chain parameters such as regional material flows and waste compositions are not included in the scope of this study.

### 2.4 Life Cycle Inventory and Evaluation Methodology

The energy and material flow data for the modeled recycling methods are derived from the original patents, published literature, and Ecoinvent v3.8 database. Due to the lack of industrial data related to the modeled processes, theoretical stoichiometry calculations are also used to derive quantities and concentrations of the chemicals used. This work is conducted using SimaPro 9.4.02 software based on the methods introduced by ISO 14041 and 14044.^57,58^ summarize the inventory used and the underlying assumptions for the CHR, THR, and PR, respectively.

### 2.5 Life Cycle Impact Assessment

The current study focuses on six different midpoint impact categories. TRACI 2.1 is used to calculate the global warming potential (kg CO_2_-eq) given its particular relevance to North American context. CED is used to calculate the energy demand (MJ). We used BEES+ to calculate the water consumption (liters) and AWARE to calculate the water footprint (m^3^). BEES+ calculates the water consumption unaffected by location, while AWARE applies regional and country-specific characterization factors to quantify the water stress in different regions or countries.^59^ USEtox, recommended for comparative chemical toxicity assessment by the European Union and the United States - Environmental Protection Agency, is used to calculate the freshwater toxicity (Comparative Toxic Units or CTU_e_).^60^ CML is used to quantify the relative contribution of a product system to the depletion of mineral resources (kg Sb-equiv) using the abiotic depletion potential (ADP) model.

### 2.6 Sensitivity Analysis

The model includes assumptions regarding the recycling efficiency of battery materials. The inventory also includes chemical input and solvent regeneration rate assumptions. A sensitivity analysis addresses the uncertainty of these assumptions and their effect on the results. The work also evaluates recycling processes in the context of existing supply chain pathways in Europe, North America, and China. It is important to note here that the developed scenarios are not exhaustive and might not be fully representative of battery supply chains, particularly in North America and Europe. A sensitivity analysis provides better clarity on (1) the comparative environmental impacts of different recycling methods and (2) the comparative environmental impacts of recycling batteries in different locations. Table 1 summarizes the variables considered in the analysis.

**Table 1:** Sensitivity Analysis Variables

Variable	Range	References
Pyrometallurgy-Lithium Recovery Efficiency (%)	[0–70]	^25^,^34^,^37^,^38^,^56^
H_2_SO_4_ input in CH and TH (kg/kg LIBP recycled)	[0.667–0.871]	^41^,^42^,^61^
H_2_O_2_ input in CH and TH (kg/kg LIBP recycled)	[0.832–1.038]	^41^,^42^,^61^
Solvent Regeneration Rate (%)	[90–100]	
Battery Waste Composition	70% Production Scrap (NMC811) and 30% Spent LIBs	
	Chemistry Composition:	
	(1) 70% NMC811, 30% NMC622 (Base scenario)	
	(2) 60% NMC811, 40% NMC622	
	(3) 80% NMC811, 20% NMC622	
Location	Base Scenarios (Refer to )	
	Sensitivity Analysis:	
	(1) North America: RFCM, SRTV, FRCC, ERCT, CAMX, NWPP, NYUP, RFCW, SRMW, SRSO, Ontario, Quebec	
	(2) Europe: Germany, Spain, Finland, Hungary, Hungary, Poland, France, United Kingdom	

## 3 Results and Discussion

The main objective of this paper is to assess the effects of recycling methods, recycling locations, and manufacturing locations on the environmental impact of future batteries. The current study focuses on global warming potential, water consumption, freshwater toxicity, and ADP impact categories. The results for the water consumption and cumulative energy demand impact categories are summarized in SI-2 sheets 1–3. For clarity and conciseness, the terms North America, Europe, and China used in this article represent different supply chain variations in each continent. shows a detailed breakdown of each scenario.

### 3.1 Environmental Impact of Recycling

As shown in Figure 2, the environmental impact of recycling varies largely across the scenarios. CHR has the highest environmental footprint for all impact categories, while truncated hydrometallurgy has the lowest footprint. The results suggest that recycling in China can increase the carbon footprint by up to 39% and the freshwater toxicity by 56% compared to those in North America and Europe. The choice of recycling method and location can reduce the carbon footprint of recycling LIBs by 87%, water consumption by 72%, freshwater toxicity by 50%, and ADP by 36% compared to the worst-case scenario. The best and worst combination changes per impact category, which highlights the need for location- and technology-specific scenario analysis. For example, for carbon footprint, the worst scenario is pyrometallurgy in China and the best case is THR in North America.

**Figure 2:**
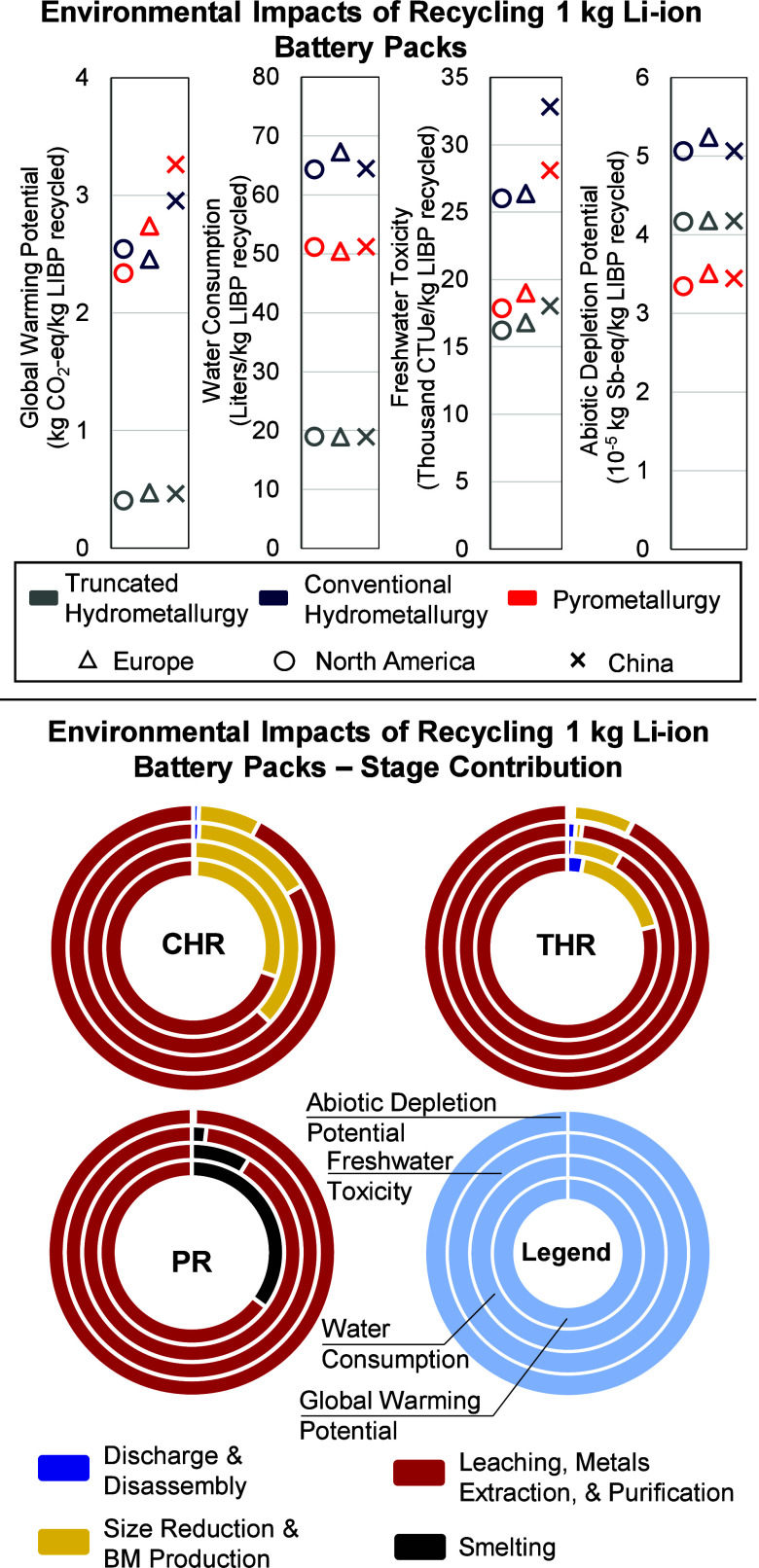
Environmental impact of recycling 1 kg of NMC811 Li-ion battery pack. The North America and Europe scenarios represent key supply chain pathways across multiple countries within each region. The specific country selections and the rationale for their inclusion are detailed in (). CHR: conventional hydrometallurgy. PR: pyrometallurgy. THR: truncated hydrometallurgy.

Several studies have assessed the environmental impact of CHR.^33,34,40–43,45,47,48^ These studies used different assumptions for location, battery waste composition, chemicals used in the leaching process, the pretreatment method, and the system boundary, making it difficult to compare the results. From those studies, the carbon footprint of recycling 1 NMC LIBP varies between 0.6 and 2.24 kg CO_2_-eq. Compared with the current work, previous studies underestimate the environmental footprint of CHR. Previous studies underestimate the CHR energy and material inputs needed. For example, one study retrieves the conventional hydrometallurgy inventory from the GREET model and ecoinvent, resulting in an overlap with the inventory of the current study for some recycling stages such as size reduction, leaching, and lithium recovery.^40^ However, this study, among others, overlooks material and energy inputs needed for other stages such as solvent extraction and crystallization (Cyanex272, electricity, and heat) and the recovery of other gypsum and sodium sulfate byproducts (calcium hydroxide, electricity). Size reduction and leaching largely contribute to the environmental impact of the process (Figure 2). Discharge and disassembly had a negligible effect. Also, electricity and chemicals contribute the most to the environmental impact of the process. The contribution of chemicals is associated with leaching and solvent extraction steps used to recover battery-grade metal sulfates. The electricity consumption from size reduction and leaching is associated with shredders, leaching reactors, and filtration equipment. Regardless of the location, the CHR has the highest water consumption, freshwater toxicity, and abiotic depletion potential. However, depending on the source of electricity, CHR can have a lower or higher carbon footprint than PR.

THR has the lowest environmental footprint, regardless of the location. The compressed flowsheet of the corresponding recycling process can explain these results. THR avoids the separation of metal sulfates, thus requiring fewer chemicals and electricity for solvent extraction, precipitation, crystallization, and solvent regeneration. Also, in contrast with CHR, the THR does not include NMP for stripping and does not include gypsum recovery. Moreover, transportation of intermediate products contributes up to 10% of the environmental impact of CH recycling but is avoided in THR (SI1 section 5 and SI2 sheet 2).

As shown in Figure 2, PR reduces the water consumption of LIB recycling by 25%, freshwater toxicity by 31%, and ADP by 34%, compared to CHR. These results are driven mainly by the lower consumption of water and chemicals in PR compared to the CHR method. However, as mentioned above, PR can have a higher or lower carbon footprint, depending on the recycling location.

Previous studies on PR reported a carbon footprint of 1.47–2.82 kg CO_2_-eq/kg battery recycled and a cumulative energy demand of 11.75–61.53 MJ/kg battery recycled.^25,27,41,46,47,61^ Although our results are similar to those reported in the literature, previous studies underestimate the environmental impact of leaching and overestimate that of smelting. For leaching, some studies do not include the chemical and electrical inputs needed to recover metal sulfates from the alloy. In this work, we include the electricity and solvent needed to separate, extract, and crystallize cobalt and nickel sulfates. Previous studies also exclude materials and energy inputs to recover Li_2_CO_3_ because they model outdated processes or patents that do not recover lithium.^62^ This work accounts for Li_2_CO_3_ recovery from flue dust via carbonated water leaching. Early PR also required significant amounts of cokes and limestone for smelting, resulting in a high carbon footprint. New PR technologies use the aluminum and carbon in the battery feed as reducing agents to deliver energy for the process.^34,37,38^ A recent study from Umicore of the updated PR calculated a carbon footprint of 2.07 kg CO_2_-eq/kg LIBP recycled.^34^ The current study shows that leaching of the alloy has the largest contribution to the environmental impact of pyrometallurgical recycling for all impact categories. The smelting contribution is higher for the carbon footprint than for other impact categories because of the resulting direct emissions. Another consideration often overlooked is that the pyro treatment removes some species, such as PVDF binder and electrolyte organics, that otherwise require dedicated process steps to remove them from the process streams directed toward battery-grade raw material synthesis.

Location also affects the LIB recycling environmental impact (Figure 2). In the current analysis, the location affects the environmental impact of the electricity grid and the chemicals used in the recycling process. As a result, recycling batteries in China reduces water consumption compared to other regions but also leads to the highest carbon footprint and freshwater toxicity. In China, coal contributes up to 60% of the Chinese electricity, which explains the high carbon footprint of recycling in China. On the other hand, SRSO and NYUP eGRID regions in the USA rely more on nuclear energy than China or Europe, causing higher water consumption.

The THR results vary marginally with the location. THR’s low energy and chemical intensities justify its low sensitivity to location. In contrast, PR and CHR show a higher sensitivity to recycling location. These results highlight opportunities to reduce the carbon footprint and toxicity of established and future PR or CHR plants. This finding is important because PR and CHR are mature, well-established technologies with high recycling capacities. On the other hand, emerging recycling methods, such as THR, are still scaling and must demonstrate the robustness of their technology at scale.

The analysis presented above compares the environmental impact of three representative recycling processes. These results reflect the absolute environmental footprint of each recycling process but do not adequately compare the potential impacts of different recycling approaches from an overall supply chain perspective. The overarching environmental advantages of battery recycling lie in reducing the reliance on primary materials. To better compare the overall battery recycling ecosystem, we must extend the system boundary to include pCAM and CAM production. The environmental impact of the new CAM reflects the material recovery efficiency of each recycling method and the primary material amounts needed to achieve the stoichiometric ratio of the NMC811 cathode. The new CAM environmental impact also depends on the environmental footprint of each recycling method, reflected through the portion of recycled materials used.

### 3.2 Environmental Impact of CAM Production

As depicted in Figure 3, the CAM environmental impact varies across the scenarios. Location has a minimal effect on the water consumption and ADP of NMC811 CAM but largely affects its carbon footprint and freshwater toxicity. The North American battery supply chain has the lowest carbon footprint and freshwater toxicity. The Chinese battery supply chain increases the carbon footprint of the new NMC811 CAM by up to 20% and the freshwater toxicity by up to 54% compared to North America and Europe. More notably, the results in Figure 3 are sensitive to the recycling method. For instance, THR scenarios have the lowest environmental footprint for all impact categories compared to PR and CHR. Only one previous study compares the environmental impacts of extracting discrete metals and recovering battery materials as a mixed salt based on the Redwood Materials recycling process.^40^ The authors show the same trend, suggesting that avoiding discrete metal recovery would reduce the environmental impact of future batteries.

**Figure 3:**
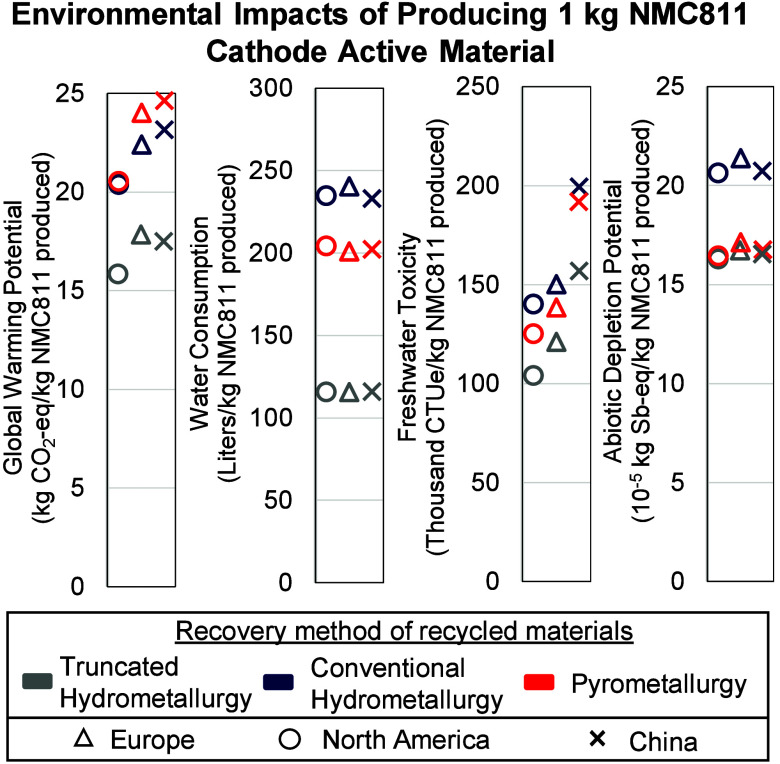
Environmental impact of producing 1 kg of NMC811 cathode active material. Note: the North America and Europe scenarios represent key supply chain pathways across multiple countries within each region. The specific country selections and the rationale for their inclusion are detailed in SI1 section 2).

Comparing Figures 3 and 4 shows a narrowing between recycling methods when focusing on the environmental impact of NMC811 CAM production. For instance, water consumption differences drop from 325% to 200% and freshwater toxicity differences from 75% to 50%. Figure 4 further suggests that the carbon footprint gap between pyro- and hydrometallurgical recycling can be negligible, which contrasts with earlier studies and with Figure 3 in this paper.^34^ The CAM recycled content in the current study exceeds projected levels.^50^ A lower recycled content would further reduce the difference between CHR, PR, and THR effects on new batteries’ environmental impact.

**Figure 4:**
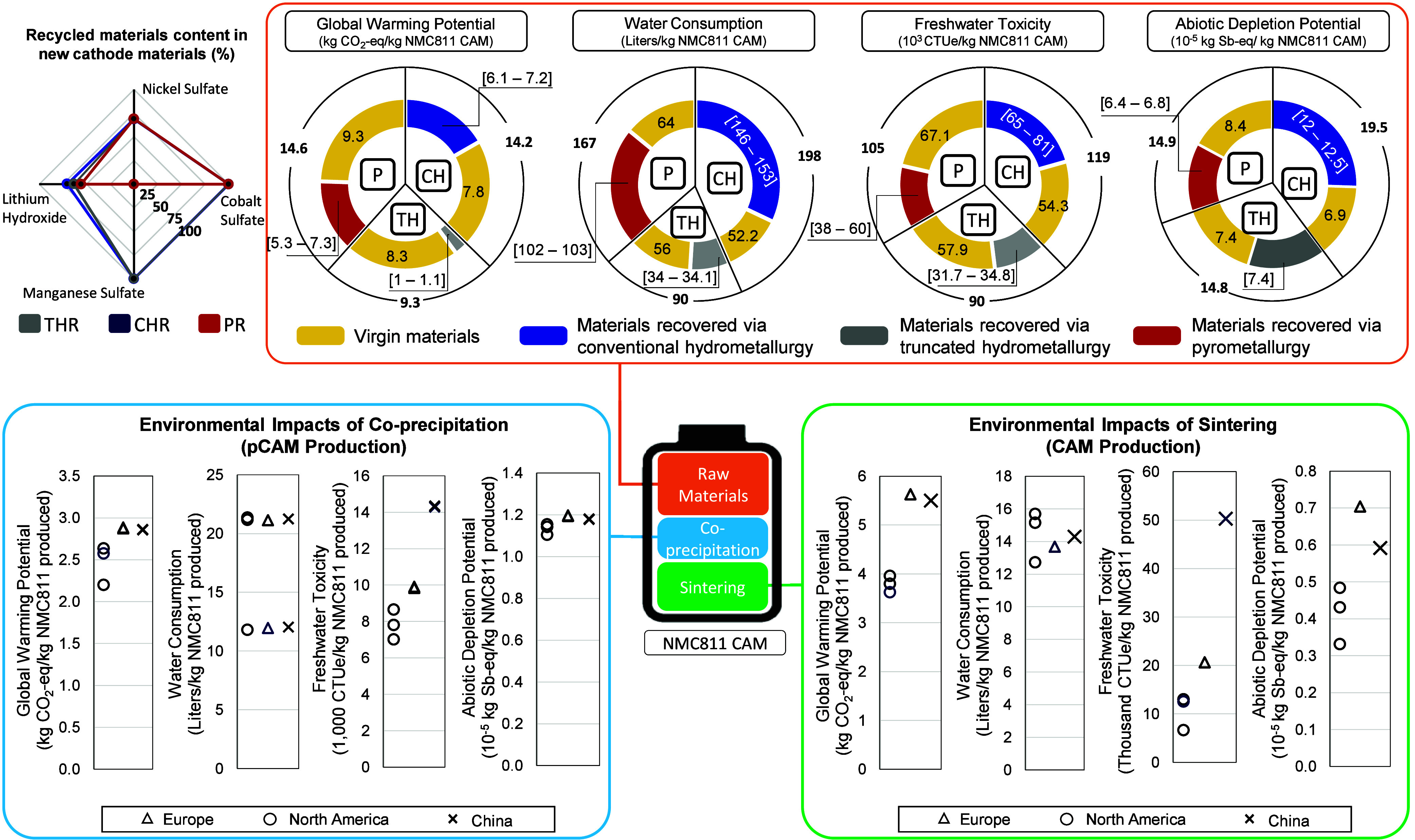
Production stages’ contribution to the environmental impact of NMC811 CAM. Note: CH, conventional hydrometallurgy; TH, truncated hydrometallurgy; P, pyrometallurgy. Spider chart (top left): recycled materials content in new CAM for each recycling method. Orange box (top): environmental impact of primary and secondary materials based on each recycling method. Blue box (bottom left): environmental impact of coprecipitation for the evaluated regions. Green box (bottom right): environmental impact of sintering for the evaluated regions. Note: the North America and Europe scenarios represent key supply chain pathways across multiple countries within each region. The specific country selections and the rationale for their inclusion are detailed in SI1 section 2.

Several studies assessed the environmental impact of cathode active materials using either primary or recycled materials but not a combination of both, which is more likely to happen in the near term. In previous work, the carbon footprint of CAM manufacturing from primary material was between 16 and 32 kg CO_2_-eq/kg NMC^46,63–66^ and secondary material, between 8.2 and 21.2 kg CO_2_-eq/kg NMC.^41,45,67^ The range of values is due to different assumptions regarding production location, CAM chemistry, and source of virgin materials. Consequently, a detailed comparison of the results with those of previous studies is challenging.

This study assesses the environmental footprint of NMC811 CAM in the US, Europe, and Asia using a mixture of primary and secondary materials. The result depends on the pCAM and CAM production locations and recycling methods. The production locations affect the sources of electricity and input chemicals. The recycling method affects the availability of recycled materials and the environmental footprint of the secondary materials.

Battery materials production is the largest contributor to the environmental impact of NMC811 CAM, followed by sintering and coprecipitation, respectively (Figure 4). Using THR secondary metals reduces the carbon footprint of battery materials by 39%, water consumption by 45%, freshwater toxicity by 43%, and ADP by 57% compared to the other scenarios. Two factors affect the environmental impact of battery materials: the content and the source of the recycled materials. For example, pyrometallurgy scenarios have the lowest recycled content and the highest carbon footprint, because more primary carbon-intensive materials are required. For instance, manganese is lost in PR, and less lithium is recovered than in THR and CHR. Thus, PR scenarios require more virgin materials makeup. The carbon footprint of virgin materials for pyrometallurgy scenarios is 22% higher than that for THR scenarios. The same applies to other impact categories and recycling methods. For example, CHR has the highest recycled content and thus requires fewer virgin materials to produce new CAM. However, in this case, the recycled materials from CHR have the highest water consumption due to the water-intensive recycling process (Figure 2).

Notably, recycling does not have the same benefits for all materials. For example, PR scenarios require 100% primary manganese, compared to 0% in the TH and CHR scenarios. A 100 percentage points (pp) difference in primary manganese content exhibits a negligible change in the environmental footprint of primary materials for all impact categories. This result is caused by the low manganese content in the NMC811 cathode material and the low environmental impact of primary manganese compared to other battery materials such as nickel or lithium. In the same context, all recycling methods lead to the same recycled cobalt content in the final CAM. Although the cobalt recycled via PR has a higher environmental impact than CHR (72% higher GWP, 57% higher water consumption, 28% higher freshwater toxicity, and 20% higher ADP), the source of cobalt has a negligible effect on the CAM environmental impact due to its low content in the NMC811 composition. By comparison, the virgin lithium carbonate content in PR scenarios is 7 pp higher than that of TH and 14 pp higher than that of CHR. The additional primary lithium increases the carbon footprint, water consumption, freshwater toxicity, and ADP of primary materials by 20%, 23%, 24%, and 22%, respectively. These results further underscore the importance of comparing recycling methods based on their contribution to offsetting primary materials needed to produce the prevailing cathode material, in this case NMC811. SI2 summarizes the environmental impact of recovered battery materials from each recycling method.

CAM production is the second largest contributor to the environmental impact of NMC811 CAM, accounting for up to 30% of the total environmental impact. The sintering step in the CAM production process consumes large amounts of electricity. Therefore, the CAM production environmental footprint varies with the location. Sintering in the US instead of China can reduce the carbon footprint by 2 kg CO_2_-eq/kWh NMC811 and the freshwater toxicity by 45,000 CTUe/kWh NMC811 due to the different electricity grid mix discussed above.

### 3.3 Sensitivity Analysis

Sensitivity analysis (SA) is helpful to assess the reliability of specific assumptions and identify the most critical factors affecting the results. The detailed sensitivity analysis results for recycling, primary materials production, pCAM production, and CAM production are summarized in SI2 sheets 6–10. The sensitivity analysis results for recycling and CAM production are discussed in this section.

The SA shows that the battery waste composition and H_2_O_2_ input assumptions have a negligible effect on the environmental impacts of recycling and CAM production. In contrast, the results are sensitive to the lithium recovery efficiency, sulfuric acid input, and solvent regeneration rate.

The CAM environmental impact is affected by the lithium recovery efficiency. This study assumes a lithium recovery efficiency of 70%, as claimed by certain recyclers like Umicore.^34,68^ Not recovering lithium in pyrometallurgy increases the demand for primary lithium. Thus, not recovering lithium increases the carbon footprint and water consumption of new CAM by 12%, the toxicity by 19%, and the ADP by 24%.

The amount of H_2_SO_4_ consumed in the leaching process varies in the literature. Results suggest that the environmental impact of THR, and consequently new CAM, is affected by the H_2_SO_4_ input, mainly affecting water consumption, toxicity, and ADP.

Solvent regeneration during Co and Ni extraction affects the environmental impact of CHR and PR. Previous studies assumed a 100% recovery efficiency. Assuming 100% solvent regeneration underestimates the recycling carbon footprint and water consumption by up to 45% and 38%, respectively. The same assumption underestimates the CAM carbon footprint and water consumption by 14% and 25%, respectively. These results highlight the importance of maximizing the solvent recovery.

The specific recycling and production supply chain scenarios evaluated in this study show that location affects the final result with North America leading to the lowest environmental impact. The sensitivity analysis results show that assuming the same location for all recycling and production stages, as in previous studies, largely affects the results. Using such generic assumptions can overestimate the environmental impact of recycling by up to 41% (39% in NA; 41% in EU) and underestimate it by up to 44% (44% in NA; 34% EU). Additional statistical analysis shows that ADP and water consumption are significantly larger in North America compared to Europe (Welch *t* test, *p* < 0.05). However, a *t* test could not demonstrate the same for carbon footprint and freshwater toxicity (*p* > 0.05).

### 3.4 Policy Implications

This paper assesses and compares the environmental footprint of three LIB recycling methods and their effect on future CAM. The average modern EV uses a 70 kWh NMC811 battery pack that contains 90 kg CAM.^69^ After comparing the best- and worst-case scenarios, the selection of the locations of production stages and the source of recycled materials can reduce the environmental impact of the 70 kWh NMC811 battery pack by 792 kg of CO_2_-eq, 11,355 L of water, 8,600,695 CTUe, and 4,580 mg of Sb-eq. In particular, the specific scenarios evaluated in this study suggest that truncated hydrometallurgy in North America leads to the lowest environmental impact of battery recycling and CAM production, compared to other recycling methods and locations. These results highlight opportunities to minimize the environmental impact of battery recycling, maximize the reusability of battery materials, and minimize the footprint of future battery CAM.

#### 3.4.1 Battery Recycling Methods

The current study results have crucial policy implications. Streamlining battery recycling and CAM production exhibits a high battery materials recovery efficiency and the lowest recycling environmental impact. The recycling industry is growing rapidly in the US and Europe, largely incentivized and supported by tax credits and loans.^70–72^ PR and CHR remain the dominant recycling methods, while newer THR is used by a few companies with a current capacity of 30,000 tons/year, merely contributing to 10% of the US recycling capacity in 2030.^70,73,74^ Future policies and incentives should support novel recycling projects that enable circularity, reduce the environmental footprint of future batteries, and contribute to reshoring of the battery supply chain through vertically integrated recycling and production processes.

The current work also evaluates new pyrometallurgical technologies that are energy efficient, recover lithium, and can match the Co and Ni recovery efficiency of CHR. PR has the same potential as CHR in reducing the carbon footprint of future CAM and is more efficient in reducing their water consumption, freshwater toxicity, and abiotic depletion potential. Of note, these results are based on the prevailing battery waste composition and the final battery chemistry. Additionally, pyrometallurgy is less complex than hydrometallurgy, has a higher tolerance for mixed battery chemistries, and can handle large battery waste volumes at once.^75^ However, in pyrometallurgy, several battery elements are lost in the slag and other components such as plastics, aluminum, and graphite act as reducing agents. Despite its advantages, PR has an inherent disadvantage in meeting the EU regulations recycled mass threshold, requiring recyclers to recover 70% of the battery weight by 2030, along with metal-specific recovery regulations.^12,15^ These regulations support and incentivize the development of alternative recycling methods to maximize the recovery efficiency of different battery components. The same regulations can limit the growth and adoption of PR. This work does not diminish the importance of cutting the recycling environmental impact or recovering materials such as aluminum, copper, and manganese in the case of CHR. However, if the primary aim is to recover battery materials and reduce future battery environmental impact, this work offers a different perspective by comparing recycling methods as a source of battery materials rather than an end-of-life management option.

#### 3.4.2 Battery Recycling and Production Location

Location largely affects the environmental footprint of recycling and CAM production. In the scenarios evaluated in this study, battery recycling and production in North America lead to the lowest carbon footprint, freshwater toxicity, and resource depletion potential, followed by Europe. In this context, multiple US and European regulations incentivize and support the reshoring of battery manufacturing and recycling. The IRA offers tax credits for EVs that use battery materials produced in the US or in free trade partner countries.^7,8^ Such requirements support recycling growth in North America and Europe to ensure IRA compliance. Several other state-level policies and incentives also attract CAM producers, cell manufacturers, and battery recyclers.^76–78^ In Europe, the EU Critical Raw Materials Act aims to expand the battery supply chain by reducing administrative burdens, streamlining permitting, and coordinating strategic stockpiling.^79^ The European Parliament further called for classifying black mass as hazardous, restricting its export, and retaining valuable metals within Europe.^80^ This regulation indirectly prevents the downstream processing of black mass in China and reduces the potentially associated environmental impacts discussed in this study. Similar regulations have not yet been implemented in North America.

However, this work shows that reshoring the battery supply chain alone is not sufficient to minimize the environmental footprint. Sensitivity analysis indicates that recycling and CAM production impacts vary substantially by region, driven primarily by the electricity grid mix. Battery recyclers and manufacturers can reduce their environmental impact by strategically locating high-impact processing stages in regions with low-carbon electricity and utilizing renewable energy sources, such as on-site solar panels or power purchase agreements. For example, the Li-cycle’s hub-and-spoke model illustrates how locating the central hub in regions with cleaner energy grids can reduce the overall environmental footprint of recycling, given that the leaching and metal extraction stages are the dominant contributors to environmental impact.

North American and European governments have adopted financial and regulatory incentives to promote renewable energy technologies.^81^ For example, the US offers investment tax credits (ITC) and production tax credits (PTC) to encourage businesses to purchase solar systems.^82^ In this context, several US manufacturers, including Tesla, GM, and Ford, currently use solar energy to power manufacturing plants.^83–86^ As battery supply chains expand and integrate vertically, aligning location-specific factors with renewable energy policies can create environmental and economic advantages.

Furthermore, the results highlight how the origin of the chemicals and materials affect battery sustainability. European regulations require manufacturers to disclose their emissions and battery carbon footprint in a publicly accessible battery passport. Similar regulations have not been enacted in the US, but state and federal policymakers are investigating solutions for battery traceability similar to those in the European Battery Passport.^87^ Future North American regulations should consider adopting traceability standards and extending disclosures beyond carbon emissions to include other critical impact categories, such as water consumption and freshwater toxicity. Policymakers can also incentivize recyclers and manufacturers to reduce their environmental footprints by setting footprint reduction targets for these indicators.

These findings underscore the importance of aligning the technology choice, location, and regulatory design to fully realize the sustainability benefits of battery recycling.

## 4 Future Perspectives

Results from this work suggest that combining recycling and CAM production processes via THR can potentially reduce the environmental footprint of recycling and CAM production compared to alternative routes. While THR offers a reduced environmental footprint and higher margin potential, it also faces greater challenges inherent to being qualified as a pCAM or CAM producer than the lower threshold qualification of operating as a metal sulfates producer.

Pyrometallurgical recycling has improved recently with lower energy demand and improved lithium recovery from the flue dust or slag.^34,68,88^ PR’s material and energy consumption improvement is primarily attributed to the use of graphite in spent LIBs as a reducing agent.^37,38^ This approach eliminates the potential recovery of battery-grade graphite by pyrometallurgy recyclers without compromising the process energy demand. This approach also risks the environmental performance of pyrometallurgy compared to THR and CHR in the future. For instance, some hydrometallurgy recyclers have announced breakthroughs in recovering battery-grade graphite, but many challenges remain in the graphite recycling space.^89,90^ The environmental impact of CHR and PR can be reduced by increasing the solvent recovery efficiency or using alternative solvents. Future research should explore the efficiency and environmental impact of these alternative solvents.

Of note, the calculations used in this study incorporate higher recycled content than the EU and China battery guideline 2030 objectives.^91^ While future policies should prioritize maximizing the recycled materials content to achievable limits, primary materials are expected to remain the main source of metal supply, contributing up to 87% by 2033.^73,92^ Further research is needed to address the following questions. “What is the effect of virgin metals provenance on the environmental impact of new batteries?” “How does the primary materials’ source affect the efficiency of recycled materials in reducing the environmental footprint of future batteries?” Additionally, this analysis does not encompass all supply chain parameters—such as region-specific battery waste compositions or the provenance of primary materials. Future work should aim to expand battery supply chain analysis to more comprehensively capture region-specific factors that influence the environmental impact of Li-ion battery recycling and manufacturing.

By evaluating recycling methods and locations as interconnected parts of the battery value chain, this study provides actionable insights for building a more circular, low-impact, and regionally resilient battery industry.

## Supplementary Material





## Notes

Additional information on the life cycle assessment scope and assumptions, scenarios setup, LIB recycling methods, life cycle inventory, LCA results by category, and literature review (PDF)
Environmental impact of CHR, environmental impact of THR, environmental impact of PR, recycled content in CAM for each scenario, environmental impact of battery materials in each scenario, sensitivity analysis, and environmental impact of recycled materials (XLSX)
