# Supplementary material for: Life Cycle Assessment of Lithium-Ion Battery Recycling: Evaluating the Impact of Recycling Methods and Location
Source: Environ Sci Technol. 2025 Jul 10;59(28):14432–43. doi: 10.1021/acs.est.4c13838 (PMC12288061; doi:10.1021/acs.est.4c13838)
Supplement: Supplementary file 1 [file es4c13838_si_001.pdf]

Life cycle assessment of Lithium-ion battery recycling: evaluating the impact of recycling methods and location

Francis Hanna[1], Calvin Somers[2], Annick Anctil[1*]

[1]*Department of Civil & Environmental Engineering, Michigan State University, East Lansing, Michigan, 48824, United States*
[2]*Department of Applied Engineering, Michigan State University, East Lansing, Michigan, 48824, United States*

[*]*Corresponding author:* anctilan@msu.edu

Summary: 45 pages, 6 figures, 24 tables

Table of Contents

1. Life Cycle Assessment Scope and assumptions S3

2. Scenarios Setup S4

3. LIB Recycling methods S10

3.1. Conventional hydrometallurgy S10

3.2. Truncated hydrometallurgy S10

3.3. Pyrometallurgy S11

4. Life Cycle Inventory S12

4.1. Conventional Hydrometallurgy - Material and energy inputs to recycle 1-ton NMC LIBP ... S12

4.2. Truncated Hydrometallurgy - Material and energy inputs to recycle 1-ton NMC LIBP S20

4.3. Pyrometallurgy - Material and energy inputs to recycle 1-ton NMC LIBP S25

5. Additional Results: Environmental Impacts by Category S34

6. Literature Review S36

References S43

1. Life Cycle Assessment Scope and assumptions

Table S1: Life cycle assessment scope and assumptions

**Scope**	*Starts with recycling NMC Li-ion battery packs and ends with the production of NMC811 cathode material*	**Timeframe/Year**	2025
**Functional Unit**	1 kg of recycled NMC622<br>1 kg of produced NMC811 produced	**Source of additional cobalt sulfate***	Mining in Congo (DRC) and refining in China
**Recycling methods***	Conventional Hydrometallurgy<br>Truncated Hydrometallurgy<br>Pyrometallurgy	**Source of additional nickel sulfate***	Nickel (MHP): mining in Indonesia and refining in China
**Impact categories**	Global Warming Potential (TRACI 2.1)<br>Cumulative Energy Demand (CED)<br>Water Consumption (BEES+)<br>Water Footprint (AWARE)<br>Freshwater Toxicity (USETox)<br>Abiotic Depletion Potential (CML)	**Source of additional manganese sulfate***	Mining in Australia and refining in China
**Variables**	Recycling method<br>Electricity grid<br>Transportation of intermediate products	**Source of additional lithium hydroxide***	Lithium (Spodumene): mining in Australia and refining to LiOH in China

Table S2: Bill-of-materials considered in the current analysis [1]

Category	Material	Weight (kg)		
		NMC111	NMC622	NMC811
Cell	Binder (PVDF)	5.96	3.48	6.31
	Copper (cell)	23.17	20.25	20.06
	Aluminum (cell)	13.13	11.50	11.45
	LiPF6	4.55	4.01	3.96
	Ethylene carbonate	12.70	11.19	11.05
	Dimethyl carbonate	12.70	11.19	11.05
	Polypropylene	2.17	1.85	2.18
	Polyethylene	0.47	0.40	0.48
	Carbon black	2.61	2.21	4.99
	Polyethylene terephthalate	0.62	0.55	0.56
	Graphite	63.67	62.52	64.60
	Lithium	9.53	7.94	6.56
	Nickel	25.84	38.49	43.01
	Manganese	20.82	12.02	5.03
	Cobalt	26.34	12.88	5.39
	Iron	-	-	-
	Phosphorus	-	-	-
	Oxygen	42.90	34.94	29.82
Module	Copper (module)	0.43	0.43	0.43
	Aluminum (module)	12.48	11.32	11.42
	Polyethylene	0.13	0.13	0.13
	Insulation (module)	0.11	0.11	0.11
	Electronic parts (module)	1.12	1.12	1.12
Pack	Copper (pack)	0.09	0.09	0.09
	Aluminum (pack)	31.09	29.52	29.56
	Steel	1.98	1.76	1.78
	Insulation (pack)	0.99	0.94	0.94
	Coolant	8.58	8.65	8.47
	Electronic parts (pack)	4.43	4.22	4.22
Total		328.61	293.30	284.75
	Specific Energy (Wh/kg)	214.80	240.70	247.90
	Energy (kWh)	70.59	70.60	70.59

Table S3: Fate and recovery assumptions of different battery pack components for conventional hydrometallurgy, truncated hydrometallurgy, and pyrometallurgy

Material	Hydrometallurgy	Hydro-to-Cathode	Pyrometallurgy
**Binder (PVDF)**	Waste	Waste	Incinerated
**Copper (cell)**	Partially recovered in leaching and size reduction step (via screening and size separation) . Processing for targeted end-use is not in the scope of this study		Recovered during leaching. Processing for targeted end-use is not in the scope of this study.
**Aluminum (cell)**	Partially recovered in leaching and size reduction step (via screening and size separation) . Processing for targeted end-use is not in the scope of this study		Lost; Used in smelter as reducing agent
**LiPF6**	Waste		Incinerated
**Ethylene carbonate**	Waste		Incinerated
**Dimethyl carbonate**	Waste		Incinerated
**Polypropylene**	Waste		Incinerated
**Polyethylene**	Waste		Incinerated
**Polyethylene terephthalate**	Partially recovered after crushing via screening and size separation. Processing for a targeted end-use is not in the scope of this study.		Incinerated
**Graphite**	Recovered at a lower-grade; not reusable in Li-ion batteries	Recovered at a lower-grade; not reusable in Li-ion batteries	Lost; Used in smelter as reducing agent
**Lithium**	Recycled		
**Nickel**	Recycled		
**Manganese**	Recycled	Recycled	Lost in slag
**Cobalt**	Recycled		
**Copper (module)**	Partially recovered in leaching and size reduction step (via screening and size separation) . Processing for targeted end-use is not in the scope of this study		Recovered during leaching. Processing for targeted end-use is not in the scope of this study.
**Aluminum (module)**	Partially recovered in leaching and size reduction step. Processing for appropriate end-use is not in the scope of this study		Lost; Used in smelter as reducing agent
**Polyethylene**	Waste	Waste	Incinerated
**Insulation (module)**	Waste	Waste	Incinerated
**Electronic parts (module)**	Waste	Waste	Lost in slag
**Copper (pack)**	Recovered during dismantling. Processing for appropriate end-use is not in the scope of this study		
**Aluminum (pack)**	Recovered during dismantling. Processing for appropriate end-use is not in the scope of this study		
**Steel**	Recovered during dismantling. Processing for appropriate end-use is not in the scope of this study		
**Insulation (pack)**	Waste		
**Coolant**	Waste		
**Electronic parts (pack)**	Waste		

2. Scenarios Setup

The current work aims to evaluate and compare Li-ion battery recycling methods within the current supply chain context, specifically accounting for prevailing battery waste composition, final cathode material outputs, and varying geographic location of recycling stages. To achieve this goal, we use scenarios reflecting existing battery recycling and manufacturing pathways in different regions. The scenarios are not exhaustive and might not be fully representative of battery supply chains, particularly in North America and Europe. This scenario analysis is followed by a sensitivity analysis to better understand the impact of recycling method and location.

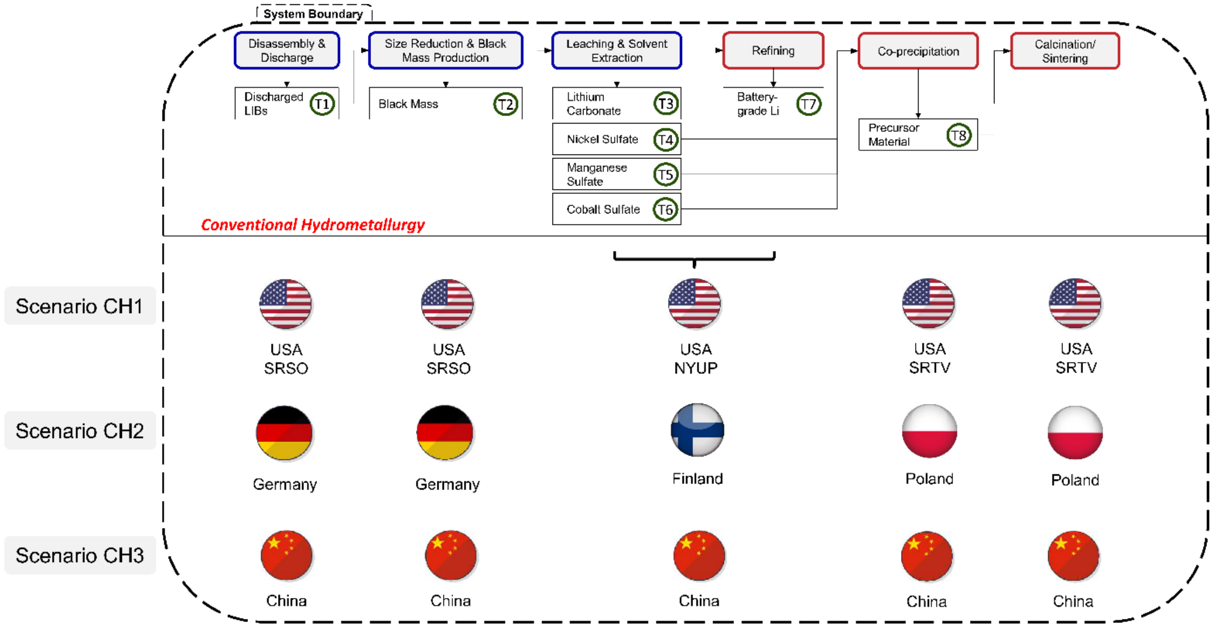

Figure S1: Conventional Hydrometallurgy - Scenarios

Table S4: Rationale for conventional hydrometallurgical recycling scenarios development

Region	Stage	Location	Rationale and Additional Information	References
North America	Disassembly and Discharge	USA SRSO: Includes the states of Alabama, Georgia, and parts of Florida	The conventional hydrometallurgy process is particularly modelled after Li-cycle's patented process. Li-cycle has an operational spoke in Alabama. Other companies with similar scopes include Call2Recycle and Full Circle Lithium	2
	Size Reduction	USA SRSO: Includes the states of Alabama, Georgia, and parts of Florida		
	Leaching & Solvent Extraction	USA NYUP: Includes the state of New York	This location is assumed following Li-cycle's intension at the time of the study to develop their first hub in Rochester, NY.	2
	Co-precipitation	USA SRTV: Includes the states of Kentucky,	Several companies currently operate and are expected to operate in the future	2

		Tennessee, and part of Mississippi	within this region including Ascend Elements, LG Chem & GM, 6K Energy, and Blue Oval SK.	
	Calcination/Sintering	USA SRTV: Includes the states of Kentucky, Tennessee, and part of Mississippi		2
Europe	Disassembly and Discharge	Germany	Germany has one of the largest EV markets, is the largest EV producing country in Europe. Over the past 3 years, Germany is the leading country in EV sales in Europe (number of electric cars registered). At the same time, several companies in Germany are involved in battery pretreatment, including collection, discharge, and disassembly such as Volkswagen, Fortum, and Duesenfeld.	3-5
	Size Reduction	Germany		
	Leaching & Solvent Extraction	Finland	Harjavalta Finland hosts Fortum, the largest hydrometallurgical recycling facility in Europe.	6
	Co-precipitation	Poland	Currently, Poland is the only country in Europe that includes operational plants manufacturing pCAM and CAM. The main companies operating in Poland include Umicore and Ascend Elements.	7, 8
	Calcination/Sintering	Poland		
China	All Stages	China has a well-established recycling and battery manufacturing industry. Due to limited information on the regional distribution of facilities, we model all stages using the national average electricity mix to represent typical conditions and ensure consistency across the supply chain.		

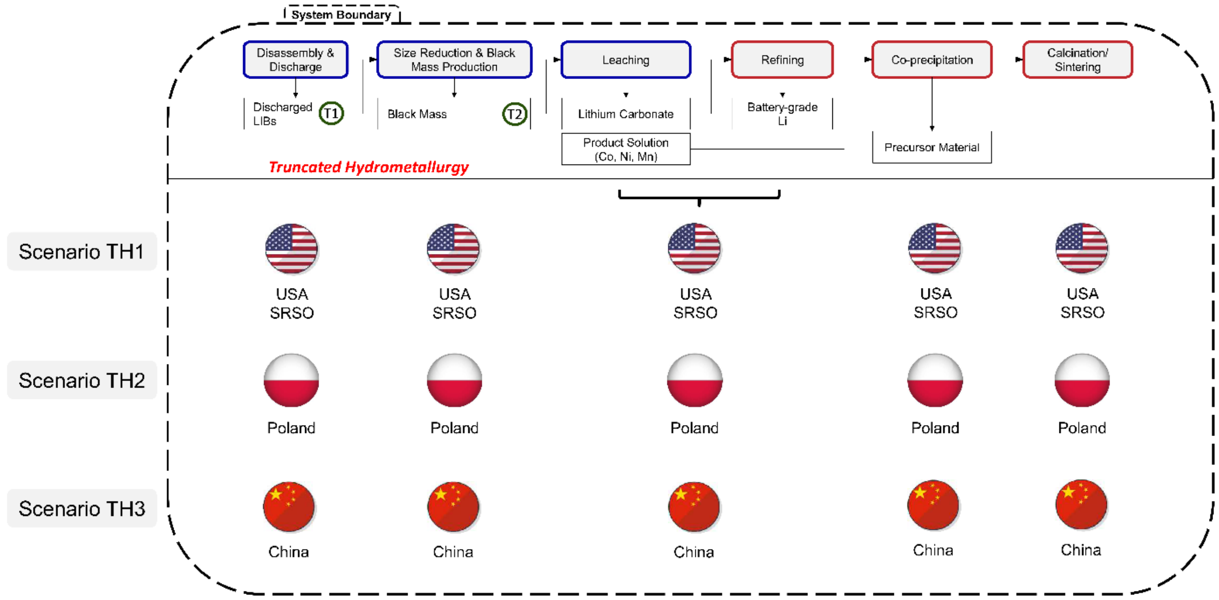

Figure S2: Truncated Hydrometallurgy – Scenarios

Table S5: Rationale for truncated hydrometallurgical recycling scenarios development

Region	Stage	Location	Additional Information	References
North America	All Stages[1]	USA SRSO: Includes the states of Alabama, Georgia, and parts of Florida	The truncated hydrometallurgy process in this study is developed based on the patent of Ascend Elements, which is currently operating in Covington, GA.	2
Europe	All Stages[1]	Poland	The truncated hydrometallurgy process in this study is developed based on the patent of Ascend Elements. Currently, the only truncated hydrometallurgy process in Europe is being developed by Ascend Elements in Poland.	7
China	All Stages[1]	China has a well-established recycling and battery manufacturing industry. Due to limited information on the regional distribution of facilities, we model all stages using the national average electricity mix to represent typical conditions and ensure consistency across the supply chain.		

[1]*Given the nature of the process, truncated-hydrometallurgy, all stages are in the same location.*

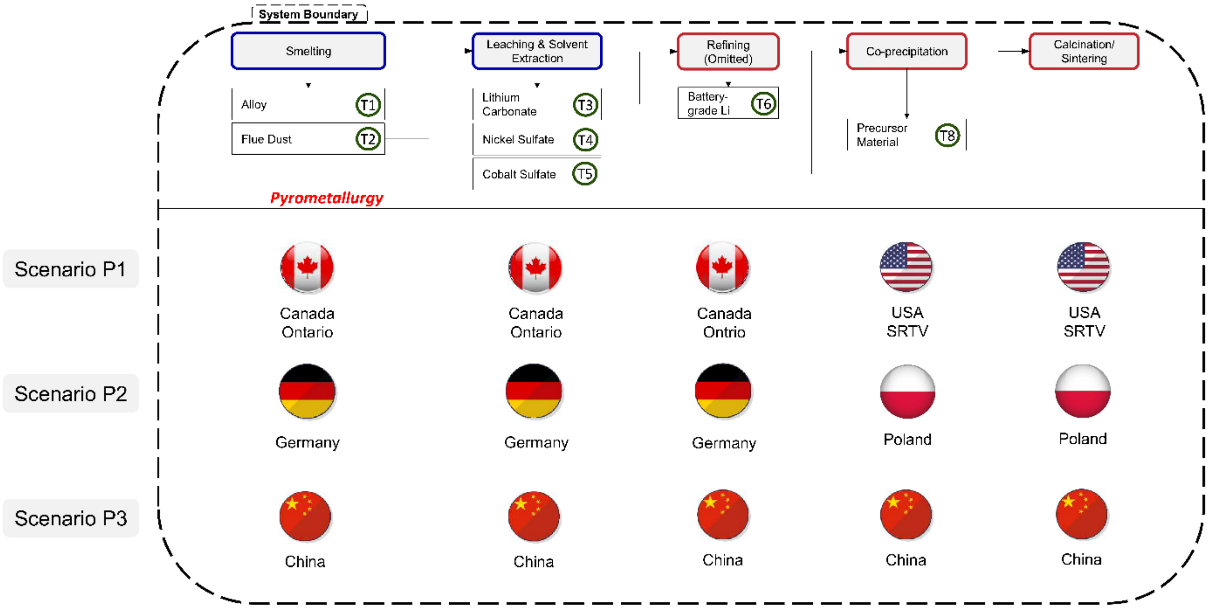

Figure S3: Pyrometallurgy – Scenarios

Table S6: Rationale for pyrometallurgical recycling scenarios development

Region	Stage	Location	Additional Information	References
North America	Disassembly and Smelting	Ontario, CA	Glencore, located in Greater Sudbury, Ontario (CA), is the largest commercial pyrometallurgical recycling company in North America, controlling approximately 60% of the North American pyrometallurgy recycling capacity.	2
	Leaching & Solvent Extraction	Ontario, CA		2

	Co-precipitation	USA SRTV: Includes the states of Kentucky, Tennessee, and part of Mississippi	Several companies currently operate and are expected to operate in the future within this region including Ascend Elements, LG Chem & GM, 6K Energy, and Blue Oval SK.	2
	Calcination/Sintering	USA SRTV: Includes the states of Kentucky, Tennessee, and part of Mississippi		2
Europe	Disassembly and Smelting	Germany	We model the pyrometallurgical recycling process following Umicore's latest process developments. Ideally, this stage should be located in Belgium. *Due to the lack of information on the 2025 electricity grid in Belgium, we assume these stages to take place in Germany.*	-
	Leaching & Solvent Extraction	Germany		
	Co-precipitation	Poland	The truncated hydrometallurgy process in this study is developed based on the patent of Ascend Elements. Currently, the only truncated hydrometallurgy process in Europe is being developed by Ascend Elements in Poland.	7, 8
	Calcination/Sintering	Poland		
China	All Stages	China has a well-established recycling and battery manufacturing industry. Due to limited information on the regional distribution of facilities, we model all stages using the national average electricity mix to represent typical conditions and ensure consistency across the supply chain.		

3. LIB Recycling methods

3.1. Conventional hydrometallurgy

This study models hydrometallurgy using a patented commercial process [9]. First, the spent battery packs are disassembled and discharged. The disassembled batteries undergo a mechanical treatment process, including recovery of ferrous elements via magnetic separation, and size-based classification. Mechanical treatment generates 'black mass', an intermediate product comprising the cathode active material (which contains most of the nickel, manganese, cobalt, and lithium), the anode (usually graphite), and some impurities. The black mass is leached using hydrogen peroxide (reducing agent) and sulfuric acid. The transition metals in the cathode material are soluble and the graphite is not, allowing separation of the two streams. The graphite is filtered off and the metal sulfate solution loaded with the target metals undergoes a series of unit operations to recover manganese, cobalt, nickel, and lithium. Manganese carbonate is isolated through precipitation, while nickel and cobalt sulfate are recovered via solvent extraction and crystallization. Finally, lithium carbonate is recovered via precipitation. The process also recovers other by-products like copper, aluminum, gypsum, and sodium sulfate. The extracted metal sulfates and lithium carbonate are used in the following steps to produce the precursor and final cathode materials. This paper uses a representative flowsheet for the conventional hydrometallurgical route, but many possible variants exist under the broad label of hydrometallurgy.

3.2. Truncated hydrometallurgy

The spent batteries undergo the same preliminary treatment as conventional hydrometallurgy. Batteries are disassembled, discharged, shredded, and sieved to obtain the black mass and other byproducts, such as plastics, steel, aluminum, and copper, using physical separation techniques that leverage differences in magnetic properties, density, and size. The black mass is leached using hydrogen peroxide and sulfuric acid, and next, lithium carbonate is recovered from the leaching solution. In contrast with conventional hydrometallurgy, metal sulfates are not extracted and separated following leaching. Instead, they remain together in the aqueous sulfate solution, and their composition is measured (TGA) and adjusted to meet the stoichiometry requirements of the final ternary metal product (NMC811 for our study). The target metal ratio is achieved by adding the appropriate quantity of nickel sulfate, manganese sulfate, and/or cobalt sulfate from external, often primary, sources. After composition adjustment, the metal sulfates are coprecipitated to produce the precursor material NMC-$(OH)_2$. Finally, the precursor is lithiated to produce CAM. The process flow diagram for this recycling method is developed using the commercialized process patent [10]. Numerous recyclers are pursuing a similar approach, choosing to avoid unnecessary materials separation steps that ultimately are going to be recombined again.

3.3. Pyrometallurgy

The modeled pyrometallurgical process reflects the latest industry practices and is developed based on patents and published literature [11-13]. First, Li-ion batteries packs are disassembled to recover the casing materials such as aluminum, copper, and steel. The disassembled Li-ion batteries then undergo a smelting step, producing a slag, an alloy, and flue dust. Here we assume that the aluminum fraction of the casing is included in the smelting charge, in line with the used patent to model the process [11, 12]. The alloy is leached using sulfuric acid and hydrogen peroxide. Next, cobalt sulfate and nickel sulfate are recovered via solvent extraction and crystallization. In the smelting process, lithium is collected in the flue dust. The latter undergoes carbonated water leaching to recover lithium carbonate. Pyrometallurgy typically consumes the graphite (anode material), but uses it beneficially as an energy source and to support the carbothermal reduction process. Although hydrometallurgy recovers graphite in theory, reintroducing the graphite into battery applications faces significant challenges, and thus the additional processing of the graphite in hydrometallurgical flowsheets must also be considered for an equitable comparison.

4. Life Cycle Inventory

4.1. Conventional Hydrometallurgy - Material and energy inputs to recycle 1-ton NMC LIBP

Table S7: Material and energy inputs for conventional hydrometallurgical recycling

Reference Product	Amount (per ton NMC111 LIBP)	Calculation and Reference	Processes Used in SimaPro
**Dismantling and Discharging**			
Deionized Water	39 kg	39 kg/metric ton of battery input [14]	Water, deionised [15]| market for water, deionised | Cut-off, U
Sodium Chloride	4 kg	4 kg/metric ton of battery input [14]	Sodium chloride, powder (GLO)| market for | Cut-off, U
**Crushing and Drying**			
Deionized Water	1.03 m^3	1.03 m^3 water/m^3 of fed Li-ion battery pack [9]	Water, deionised [15]| market for water, deionised | Cut-off, U
Electricity	135.46 kWh	158 kWh/ton feed [14, 16]	Electricity, medium voltage | market group for | Cut-off, U
**Stripping**			
NMP	Total input = 76.8 kg<br><br>Net input = 7.68 kg (Assuming a 90% regeneration rate)	1 m^3/ton of influent solids [9]	N-methyl-2-pyrrolidone | market for | Cut-off, U
**Black Mass Filtering**			
Electricity	29.53 kWh	The machinery inventory provided in the EverBatt model was used. The design capacity of a filter press is 0.60 t/h and the electrical power at design capacity is 29.828 kW [17]	Electricity, medium voltage | market group for | Cut-off, U
**Black Mass Leaching**			

Sulfuric Acid	[667 - 871] kg	Three methods cross-checked:<br>- 1st method is by stoichiometry based on a 2M concentration.<br>- 2nd method based on a rate retrieved from the literature 1.08 kg/kg cell recycled; this is equivalent to 878 kg/ton battery feed [17]<br>- 3rd method based on a rate reported in the literature 871 kg/ton battery feed [14]<br>The three approaches led to close results. The stoichiometric calculation with 10% excess acid is used (666.38 kg).	Sulfuric acid | market for sulfuric acid | Cut-off, U
Hydrogen Peroxide	[832 – 1038] for 3% H_2O_2<br><br>40.8 kg for 50% H_2O_2	Two methods cross-checked:<br>- 1st method is by stoichiometry. The referenced patent suggests a use of 30 g/L concentration. The final number (832 kg) is adjusted to account for dilution of 50% H_2O_2 as represented in Ecoinvent.<br>- 2nd method based on a rate retrieved from the literature 1.038 kg/kg battery pack recycled; this is equivalent to (1038 kg) kg/ton battery feed [17]	Hydrogen peroxide, without water, in 50% solution state | market for hydrogen peroxide, without water, in 50% solution state | Cut-off, U
Deionized Water	3,776 kg	Another way is used where water is estimated from the two stoichiometric calculations for H_2SO_4 and H_2O_2.<br>H_2SO_4 calculations yield a water consumption of 2,989.21 L.<br>H_2O_2 calculations yield a water consumption of 791.17 L.	Water, deionised [15]| market for water, deionised | Cut-off, U

Electricity for Leaching	17.72 kWh	Retrieved from [14]	Electricity, medium voltage | market group for | Cut-off, U
Electricity for Filtration	10 kWh	The machinery inventory provided in the EverBatt model was used. The design capacity of a filter press is 0.60 t/h and the electrical power at design capacity is 29.828 kW [17] In this step, 201.68 kg is set to be filtered. This includes graphite and carbon black.	Electricity, medium voltage | market group for | Cut-off, U
**Flotation**			
Water	605.04 kg	Retrieved from [18]	Tap water, at user/US- US-EI U
Electricity	37.60 kWh	The machinery inventory provided in the EverBatt model was used. The design capacity of a filter press is 0.80 t/h and the electrical power at design capacity is 149.14 kW [17] In this step, the input into the flotation step weighs 201.68 kg.	Electricity, medium voltage | market group for | Cut-off, U
**Metal Sulfates Extraction – Manganese**			
Electricity Mn Precipitation	7.602 kWh	Calculated based on the rate 125.70 kWh/t Mn [19]	Electricity, medium voltage | market group for | Cut-off, U
Sodium Carbonate	122 kg	$MnSO_{4(aq)} + Na_2CO_{3(s)} \rightarrow MnCO_{3(s)} + Na_2SO_{4(aq)}$ Theoretical stoichiometry calculations were used to estimate the mass of Na_2CO_3 needed	Soda ash, light, crystalline, heptahydrate [20]| market for | Cut-off, U
**Metal Sulfates Extraction – Cobalt**			
Solvent (15% Cyanex272) $V_{solvent}$	4,500 Liters	Assuming a A:O ratio of 1 [21], the solvent volume should be equal to the aqueous solution volume. To calculate the volume of the incoming product solution, an average density of 1 kg/m^3 is assumed.	
Solvent net volume (15% Cyanex272) $V_{solvent}$	225 Liters	This is based on the required solvent volume and the regenerated solvent volume. A regeneration factor of 0.95 is assumed [22].	
Electricity Co Crystallization	9.056 kWh	Calculated based on the rate 115.29 kWh/t Co [19]	Electricity, medium voltage | market group for | Cut-off, U
Electricity (Reaction & Workup)	140 MJ (38.89 kWh)	$0.7 \times m_{Product}$ $m_{Product} = 200$ kg ($CoSO_4$)	Electricity, medium voltage | market group for | Cut-off, U
Steam (Reaction & Workup)	240 kg	$1.2 \times m_{Product}$ $m_{Product} = 200$ kg ($CoSO_4$)	Steam, in chemical industry | market for steam, in chemical industry | Cut-off, U
**Metal Sulfates Extraction – Nickel**			
Solvent (15% Cyanex272)	4,300 Liters	Assuming a A:O ratio of 1 [21], the solvent volume should be equal to the	

		aqueous solution volume. To calculate the volume of the incoming product solution, an average density of 1 kg/m^3 is assumed.	
Solvent net volume (15% Cyanex272) $V_{solvent}$	215 Liters	This is based on the required solvent volume and the regenerated solvent volume. A regeneration factor of 0.95 is assumed [22].	
Electricity Ni Crystallization	4.67 kWh	Calculated based on the rate 60.62 kWh/t Ni [19]	Electricity, medium voltage | market group for | Cut-off, U
Electricity (Reaction & Workup)	137.9 MJ (38.31 kWh)	0.7 x $m_{Product}$ $m_{Product}$ = 197 kg ($NiSO_4$)	Electricity, medium voltage | market group for | Cut-off, U
Steam (Reaction & Workup)	236.4 kg	1.2 x $m_{Product}$ $m_{Product}$ = 197 kg ($NiSO_4$)	Steam, in chemical industry | market for steam, in chemical industry | Cut-off, U
**CuS Extraction**			
Sodium Sulfide	4.05 kg	$CuSO_{4(aq)} + Na_2S_{(s)} \rightarrow CuS_{(s)} + Na_2SO_{4(aq)}$ Theoretical stoichiometry calculations were used to estimate the mass of Na_2CO_3 needed [23]	Sodium sulfide [20]| market for | Cut-off, U
Electricity (Filtration)	0.37 kWh	The machinery inventory provided in the EverBatt model was used. The design capacity of a filter press is 0.80 t/h and the electrical power at design capacity is 149.14 kW [17] In this step, the input into the flotation step weighs 7.46 kg. The CuS precipitated weight was estimated based on the Battery BOM and copper recovery rate of 90% as per [9]	Electricity, medium voltage | market group for | Cut-off, U
**Al/Fe Extraction**			
Sodium Hydroxide	15.95 kg	Estimated based on theoretical stoichiometric calculations. The reaction equation was retrieved from the original patent [9]. The result is 13.42 kg NaOH/kg Al. This is equivalent to 26.84 kg NaOH in 50% H_2O per kg Aluminum reacted.	Sodium hydroxide, without water, in 50% solution state | market for | Cut-off, U
Electricity (Precipitation)	0.676 kWh	There was no data on the precipitation electricity consumption. As such, the electricity consumption rate was assumed to be the same as Mn precipitation (i.e., 125.70 kWh/ton Mn).	Electricity, medium voltage | market group for | Cut-off, U
Electricity (S/L Separation)	0.77 kWh	The electricity consumption for S/L separation was estimated using machinery data for the filter press	Electricity, medium voltage | market group for | Cut-off, U

		from EverBatt model [17]. The design capacity of a filter press is 0.80 t/h and the electrical power at design capacity is 149.14 kW. In this step, 15.55 kg of $Al(OH)_3$ will be separated/filtered.	
	**Sodium Sulfate**		
Electricity (Crystallizing)	27.88 kWh	The crystallization electricity consumption was assumed to be the same as for nickel sulfate. This was based on the resemblance between the solubility as a function of temperature for both Nickel and sodium Sulfate.	Electricity, medium voltage | market group for | Cut-off, U
Electricity (S/L Separation)	22.87 kWh	The electricity consumption for S/L separation was estimated using machinery data for the filter press from EverBatt model [17]. The design capacity of a filter press is 0.80 t/h and the electrical power at design capacity is 149.14 kW. In this step, 460 kg of Sodium Sulfate will be separated/filtered. This number was estimated based on the Li-cycle process description shared by Ford.	Electricity, medium voltage | market group for | Cut-off, U
	**Gypsum Extraction**		
Calcium Hydroxide	184.82 kg	Estimated based on theoretical stoichiometric calculations [24]. The stoichiometric requirement is 0.43 kg $Ca(OH)_2$ per kg Gypsum precipitated.	Lime, hydrated, loose weight| market for lime, hydrated, loose weight | Cut-off, U
Electricity (Precipitation)	12.83 kWh	The electricity consumption for precipitation was estimated using machinery data for the precipitation tank from EverBatt model [17]. The design capacity of a precipitation tank is 0.40 t/h and the electrical power at design capacity is 11.931 kW. In this step, 430 kg of gypsum will be precipitated. This number was estimated based on the Li-cycle process description shared by Ford.	Electricity, medium voltage | market group for | Cut-off, U
Electricity (Filtration)	21.37 kWh	The electricity consumption for filtration was estimated using machinery data for the filter press from EverBatt model [17]. The design capacity of a filter press is 0.80 t/h and the electrical power at design capacity is 149.14 kW. In this step, 430 kg of gypsum will be	Electricity, medium voltage | market group for | Cut-off, U

		separated/filtered. This number was estimated based on the Li-cycle process description shared by Ford.	
**Lithium Carbonate Isolation**			
Sodium Carbonate	221.41 kg	Estimated based on theoretical stoichiometric calculations [9]. This results in 7.63 kg sodium carbonate per kg Lithium precipitated.	Soda ash, light, crystalline, heptahydrate | market for | Cut-off, U
Electricity (Precipitation)	6.956 kWh	Calculated based on the rate 242.29 kWh/t Li [19]	Electricity, medium voltage | market group for | Cut-off, U
Electricity (S/L Separation)	7.67 kWh	The electricity consumption for S/L separation was estimated using machinery data for the filter press from EverBatt model [17]. The design capacity of a filter press is 0.80 t/h and the electrical power at design capacity is 149.14 kW. In this step, 154.36 kg of crude lithium carbonate will be separated/ filtered.	Electricity, medium voltage | market group for | Cut-off, U
Wastewater Treatment	3821.12	The wastewater volume is calculated based on an input-output approach. After accounting for the inputs, and main product (Li_2CO_3), 3821.12 kg remains after filtration.	Wastewater, average | market for wastewater, average | Cut-off, U

Life Cycle Inventory of Organic Solution (Inventory per liter of Organic Solution)

The inventory below is estimated based on a study that looked into the separation of cobalt and nickel via solvent extraction using Cyanex272 [21]. The study consists of an experimental analysis to assess the variation of cobalt extraction efficiency using CYANEX 272 under different conditions. The authors vary the solution pH, solvent dilution, solvent saponification, and aqueous-to-organic solution ratio. According to this study, it is uncommon to use extractants in their pure form, and they are usually dissolved in an organic diluent. The table below summarizes the composition of the organic solution used, consisting of 15% Cyanex272, 5% modifier, and 80% Naphtha [21].

Table S8: Life cycle inventory of the organic solution

Reference Input	Amount	Process
Cyanex-272 (ml)	150	Refer the following section for Cyanex272 inventory
Modifier (Isodecanol) (ml)	50	Omitted
Naphtha (ml)	800	Naphtha | market for | Cut-off, U

Life Cycle Inventory of Cyanex272 Extractant [25]

Table S9: Life cycle inventory of Cyanex272 Extractant

Reference Input	Amount	Process
3-methyl-1-butanol (kg)	1.9	3-methyl-1-butanol | market for | Cut-off, U
Sodium Hypophosphite (kg)	0.46	Refer to the following section
Electricity (kWh)	1.36	Electricity, medium voltage {US}| market group for | Cut-off, U
**Output**	**Amount**	**Process**
Cyanex272 (kg)	1	Reference Product
Calcium Carbonate (kg)	0.71	Emission to air
Phosphine (kg)	0.16	Emission to water

Life Cycle Inventory of Sodium Hypophosphite [25]

Table S10: Life cycle inventory of Sodium Hypophosphite

Reference Input	Amount	Process
Soda Ash (kg)	0.75	Soda ash, light, crystalline, heptahydrate | market for | Cut-off, U
Lime (kg)	0.52	Lime, hydrated, loose weight | market for lime, hydrated, loose weight | Cut-off, U
Phosphorus (kg)	0.56	Phosphorus, white, liquid | market for | Cut-off, U
Tap Water (kg)	0.25	Tap water | market group for | Cut-off, U
**Output**	**Amount**	
Sodium Hypophosphite (kg)	1	Reference Product

Embodied emissions, energy, water footprint, and toxicity for input materials (Without Electricity Input)

Table S11: Embodied environmental impacts of input materials

Chemical/Input	GWP (kg CO_2-eq/kg of product)	Embodied Energy (MJ/kg of product)	Water footprint (AWARE) (m^3/kg of product)	Water footprint (BEES+) (Liters/kg of product)	Freshwater Ecotoxicity (CTUe)	*Abiotic Depletion Potential (kg Sb-eq)*	*Electricity Input (kWh/kg)*
**Deionized Water**	0.434 E-3	6.3 E-3	0.0449	1	13	2.67E-8	2.6 E-5 MV
**NaCl**	0.195	2.77	0.134	3.42	4.22E3	1.32E-5	0.17 MV
**NMP**	6.21	123	11.2	277	3.29E4	6.54E-5	0.416 MV
**H_2O_2**	1.05	17.4	3.08	72.6	1.26E4	1.44E-5	0.56 MV
**Na_2CO_3**	0.426	5.33	0.655	16.2	6.47E3	1.6E-5	0.0143 MV
**Na_2S**	2.78	36.8	4.63	111	4.18E4	10.5E-5	0.331 MV
**Na(OH)**	0.327	4.81	0.92	22.2	5.79E3	1.73E-5	1.314 MV
**$Ca(OH)_2$**	0.915	4.49	0.0271	0.82	224	1.17E-7	0.00636 MV
**Nitrogen Liquid (N_2)**	0.00465	72.3E-3	0.354	8.3	20.1	5.62E-8	0.563 MV
**Solvent (15% Cyanex 272)***	1.9E3	64.1E3	2.1E3	5.33E4	6.56E6	723E-5	1.36 MV

**The functional unit for the solvent is 1 m^3*

Embodied emissions, energy, water footprint, and toxicity for input materials and processes

Table S12: Embodied environmental impacts of input materials

Input/Process	Functional Unit	GWP (kg CO_2-eq/FU)	Embodied Energy (MJ/FU)	Water footprint (AWARE) (m^3/FU)	Water footprint (BEES+) (Liters/FU)	Abiotic Depletion Potential (kg Sb-eq)	Freshwater Ecotoxicity (CTUe/FU)
**Tap Water**	1 kg	0.00075	12.6E-3	0.041	1	3.16E-10	3.82
**H_2SO_4**	1 kg	0.16	3.55	0.575	13.7	10.8E-5	2.33E4
**Wastewater treatment**	1 m^3	0.543	6.68	-38.2	-895	6.48E-6	8.73E3
**Steam**	1 kg	0.0127	175E-3	0.00255	0.0774	9.11E-8	1E3
**Waste Disposal/Landfill**	1 kg	0.106	0.241	0.0086	0.256	-	778

**Electronics Waste**	1 kg	0.0507	0.816	0.00847	0.379	7.85E-7	389

4.2. Truncated Hydrometallurgy - Material and energy inputs to recycle 1-ton NMC LIBP

Table S13: Life cycle inventory of truncated hydrometallurgy recycling

Reference Product	Amount	Calculation and Reference	Processes Used in SimaPro
**Dismantling and Discharging**			
Deionized Water	39 kg	39 kg/metric ton of battery input [14]	Water, deionised | market for water, deionised | Cut-off, U
Sodium Chloride	4 kg	4 kg/metric ton of battery input [14]	Sodium chloride, powder (GLO)| market for | Cut-off, U
**Crushing and Drying**			
Deionized Water	1.03 m^3	1.03 m^3 water/m^3 of fed Li-ion battery pack [9]	Water, deionised | market for water, deionised | Cut-off, U
Electricity	135.46 kWh	158 kWh/ton feed [14, 16]	Electricity, medium voltage | market group for | Cut-off, U
**Black Mass Filtering**			
Electricity	29.53 kWh	The machinery inventory provided in the EverBatt model was used. The design capacity of a filter press is 0.60 t/h and the electrical power at design capacity is 29.828 kW [17]	Electricity, medium voltage | market group for | Cut-off, U
**Black Mass Leaching**			
Sulfuric Acid	[667 - 871] kg	Three methods cross-checked:<br>- 1st method is by stoichiometry based on a 2M concentration.<br>- 2nd method based on a rate retrieved from the literature 1.08 kg/kg cell recycled; this is equivalent to 878 kg/ton battery feed [17]<br>- 3rd method based on a rate reported in the literature 871 kg/ton battery feed [14]<br>The three approaches led to close results. The stoichiometric calculation with 10% excess acid is used (666.38 kg).	Sulfuric acid | market for sulfuric acid | Cut-off, U

Hydrogen Peroxide	[832 – 1038] for 3% H_2O_2<br><br>40.18 kg for 50% H_2O_2	Two methods cross-checked:<br>- 1st method is by stoichiometry. The referenced patent suggests a use of 30 g/L concentration. The final result for 1 ton LIBP (832 kg) is adjusted to account for dilution of 50% H_2O_2 as represented in Ecoinvent.<br>- 2nd method based on a rate retrieved from the literature 1.038 kg/kg battery pack recycled; this is equivalent to (1038 kg) kg/ton battery pack feed [17]	Hydrogen peroxide, without water, in 50% solution state | market for hydrogen peroxide, without water, in 50% solution state | Cut-off, U
Deionized Water	3,776 kg	Another way is used where water is estimated from the two stoichiometric calculations for H_2SO_4 and H_2O_2.<br>H_2SO_4 calculations yield a water consumption of 2,989.21 L.<br>H_2O_2 calculations yield a water consumption of 791.17 L.	Water, deionised | market for water, deionised | Cut-off, U
Electricity for Leaching	17.72 kWh	Retrieved from [14]	Electricity, medium voltage | market group for | Cut-off, U
Electricity for Filtration	10 kWh	The machinery inventory provided in the EverBatt model was used. The design capacity of a filter press is 0.60 t/h and the electrical power at design capacity is 29.828 kW [17] In this step, 201.68 kg is set to be filtered. This includes graphite and carbon black.	Electricity, medium voltage | market group for | Cut-off, U
**Flotation**			
Water	605.04 kg	Retrieved from [18]	Tap water, at user/US- US-EI U
Electricity	37.60 kWh	The machinery inventory provided in the EverBatt model was used. The design capacity of a filter press is 0.80 t/h and the electrical power at design capacity is 149.14 kW [17] In this step, the input into the flotation step weighs 201.68 kg.	Electricity, medium voltage | market group for | Cut-off, U
**Impurities Removal (Cu)**			
Sodium Sulfide	4.05 kg	$CuSO_{4(aq)} + Na_2S_{(s)} \rightarrow CuS_{(s)} + Na_2SO_{4(aq)}$	Sodium sulfide [20]| market for | Cut-off, U

		Theoretical stoichiometry calculations were used to estimate the mass of Na_2CO_3 needed [23]	
Electricity (Filtration)	0.37 kWh	The machinery inventory provided in the EverBatt model was used. The design capacity of a filter press is 0.80 t/h and the electrical power at design capacity is 149.14 kW [17] In this step, the input into the flotation step weighs 7.46 kg. The CuS precipitated weight was estimated based on the Battery BOM and copper recovery rate of 90% as per [9]	Electricity, medium voltage | market group for | Cut-off, U
**Impurities Removal (Al)**			
Sodium Hydroxide	15.95 kg	Estimated based on theoretical stoichiometric calculations. The reaction equation was retrieved from the original patent [9]. The result is 13.42 kg NaOH/kg Al. This is equivalent to 26.84 kg NaOH in 50% H_2O per kg Aluminum reacted.	Sodium hydroxide, without water, in 50% solution state | market for | Cut-off, U
Electricity (Precipitation)	0.676 kWh	There was no data on the precipitation electricity consumption. As such, the electricity consumption rate was assumed to be the same as Mn precipitation (i.e., 125.70 kWh/ton Mn).	Electricity, medium voltage | market group for | Cut-off, U
Electricity (S/L Separation)	0.77 kWh	The electricity consumption for S/L separation was estimated using machinery data for the filter press from EverBatt model [17]. The design capacity of a filter press is 0.80 t/h and the electrical power at design capacity is 149.14 kW. In this step, 15.55 kg of $Al(OH)_3$ will be separated/filtered.	Electricity, medium voltage | market group for | Cut-off, U
**Lithium Carbonate Recovery**			
Sodium Carbonate	221.41 kg	Estimated based on theoretical stoichiometric calculations [9]. This results in 7.63 kg sodium carbonate per kg Lithium precipitated.	Soda ash, light, crystalline, heptahydrate | market for | Cut-off, U
Electricity (Precipitation)	6.956 kWh	Calculated based on the rate 242.29 kWh/t Li [19]	Electricity, medium voltage | market group for | Cut-off, U
Electricity (S/L Separation)	7.67 kWh	The electricity consumption for S/L separation was estimated using machinery data for the filter press from EverBatt model [17]. The design capacity of a filter press is 0.80 t/h	Electricity, medium voltage | market group for | Cut-off, U

		and the electrical power at design capacity is 149.14 kW. In this step, 154.36 kg of crude lithium carbonate will be separated/ filtered.	
Wastewater Treatment	3821.12	The wastewater volume is calculated based on an input-output approach. After accounting for the inputs, and main product (Li_2CO_3), 3821.12 kg remains after filtration.	Wastewater, average | market for wastewater, average | Cut-off, U
**Composition Adjustment**			
Cobalt Sulfate<br>Nickel Sulfate<br>Manganese Sulfate	The required weight of primary materials in this stage is calculated based on the input battery waste, the chemistry of the output cathode material, and the recovery efficiency of the process.		The inventory for primary materials is retrieved from the GREET Model [26]
**Coprecipitation**			
The used inventory is retrieved from the GREET Model [1]			
**Sintering**			
The used inventory is retrieved from the GREET Model [1]			

Environmental impacts of input materials (Without Electricity Input)

Table S14: Embodied environmental impacts of input materials

Input	GWP (kg CO_2-eq/kg of product)	Embodied Energy (MJ/kg of product)	Water footprint BEES+ (L/kg of product)	Water footprint AWARE (m^3/kg of product)	Ecotoxicity (CTUe)	Abiotic Depletion Potential (kg Sb-eq)	*Electricity Input (kWh/kg)*
**Deionised Water**	0.434 E-3	6.3 E-3	1	0.0449	13	2.67E-8	2.6 E-5 MV
**NaCl**	0.195	2.77	3.42	0.134	4.22E3	1.32E-5	0.17 MV
**H_2O_2**	0.895	17.8	72.7	2.93	1.07E4	-	0.56 MV
	1.05	17.4	72.6	3.08	1.26E4	1.44E-5	0.56 MV
	0.902	16.5	261	9.41	6.56E3	-	0.56 MV
**Na_2CO_3**	0.426	5.33	16.2	0.655	6.47E3	1.6E-5	0.0143 MV
**Na_2S**	2.78	36.8	111	4.63	4.18E4	10.5E-5	0.331 MV

**Na(OH)**	0.327	4.81	22.2	0.92	5.79E3	1.73E-5	1.314 MV

Environmental impacts of input materials

Table S15: Embodied environmental impacts of input materials

**Input/Process**	**Functional Unit**	**GWP (kg CO_2-eq/FU)**	**Embodied Energy (MJ/FU)**	**Water footprint (AWARE) (m^3/FU)**	**Water footprint (BEES+) (Liters/FU)**	***Abiotic Depletion Potential (kg Sb-eq)***	**Ecotoxicity (CTUe/FU)**
**Tap Water**	1 kg	0.00075	12.6E-3	0.041	1	3.16E-10	3.82
**H_2SO_4**	1 kg	0.16	3.55	0.575	13.7	10.8E-5	2.33E4
**Wastewater treatment**	1 m^3	0.543	6.68	-38.2	-895	6.48E-6	8.73E3
**Waste Disposal/Landfill**	1 kg	0.106	0.241	0.0086	0.256	-	778
**Electronics Waste**	1 kg	0.0507	0.816	0.00847	0.379	7.85E-7	389

4.3. Pyrometallurgy - Material and energy inputs to recycle 1-ton NMC LIBP

Table S16: Life cycle inventory of pyrometallurgy recycling

Reference Product	Amount	Calculation and Reference	Processes Used in SimaPro
**Preheating, Pyrolysis & Smelting**			
Coke	Coke input is not considered in this study based on the latest Umicore patents for pyrometallurgical recycling of Li-ion batteries. According to the Umicore patents, the new smelting process does not require coke.	- 86.5 kg/kg Li-ion battery packs [27]<br>- 157 kg/ton Li-ion battery packs input [14]<br>- 400 kg/ton battery input [28]<br>- Some studies do not include any coke input [29, 30]. These studies consider pretreatment steps leading to treated cells entering the smelter. Another study, does not include coke [17, 31]. This is the documentation for the EverBatt model.<br>- No coke is needed as per the latest Umicore patent. Heat is supplied by the oxidation of Al & C in the batteries [11, 12].	-
Limestone	160 kg	- 0.86 ton/ton cobalt – This number is based on the old Umicore patent [31]<br>- 0.3 ton/ton spent battery packs [17]<br>- 0.039 ton/ton Li-ion battery packs [14]<br>- 0.083ton/ton Li-ion battery packs[28]<br>- 78.6 kg/kg spent Li-ion batteries [27]<br>- 0.35 ton/ton Li-ion battery pack assuming we want to extract Mn, Ni, and Co [12]. Note, here the batteries are shredded for easier mixing.<br>- **In this study, the limestone input is retrieved from the latest Umicore patent. In the latter, the material inputs are presented for a single smelting step conducted at a pilot scale. Consequently, the limestone input used is 80 kg for 500 kg of battery packs feed (without pretreatment) [11].**	Limestone, unprocessed | market for limestone, unprocessed | Cut-off, U
Silica/Sand	40 kg	- 150 kg/ton spent Li-ion battery [17]<br>- 92 kg/ton Li-ion battery packs [28]<br>- 200 kg/ton Li-ion battery pack assuming we want to extract Mn, Ni, and Co [12]. Note, here the batteries are shredded for easier mixing.	Silica sand [20]| market for | Cut-off, U

		- **In this study, the silica input is retrieved from the latest Umicore patent. In the latter, the material inputs are presented for a single smelting step conducted at a pilot scale. Consequently, the silica input is 20 kg for 500 kg of Li-ion battery packs feed (without pretreatment) [11].**	
Electricity	440 kWh	- 2.88 MJ/kg battery pack input [29]<br>- 4.481 MJ/kg battery pack input [14]<br>- 0.69 mmBTU/ton battery input or 1.45 mmBTU/ton cobalt – including the pretreatment and pyrolysis steps [28, 31]<br>- 8 kWh/kg treated Li-ion battery cells (from an aggregated dataset based on Batrec process) [30]<br>- 4.68 MJ/kg Li- battery pack input – including the pretreatment and pyrolysis steps[17]<br>- **Varies depending on the oxygen flow into the smelter [11].** **60 kWh/ton Li-ion battery packs feed, assuming a 77 Nm^3 O_2.** **In this study, 440 kWh/ ton Li-ion battery packs feed, assuming a 45 Nm^3 O_2.**	Electricity, medium voltage | market group for | Cut-off, U
O_2 gas flow (1.784 kg/Nm^3)	84 Nm^3	Ranges between 42 and 77 Nm^3 for 500 kg of battery feed [11].	Oxygen, liquid | market for | Cut-off, U
Direct Emissions	709 kg	On average, 1 ton of LIBPs contains 190 kg of graphite. Assuming graphite is completely burned/incinerated in the smelting process, this would release an equivalent of 709 kg of CO_2 emissions [32].	Carbon Dioxide – Emissions to air
Offgas Cleaning		Refer to following section	
**Leaching and Cu Precipitation**			
Hydrogen peroxide	305.5 kg	Estimated based on theoretical stoichiometric calculations. Assumed to be added at a concentration of 10% (v/v) with respect to H_2SO_4	Hydrogen peroxide, without water, in 50% solution state | market for hydrogen peroxide, without water, in 50% solution state | Cut-off, U
Sulfuric Acid	412 kg	Estimated based on theoretical stoichiometric calculations. The estimated number includes an additional 10%. The original concentration considered in the calculations in 2M and results in 2356 kg. However, the process in ecoinvent	Sulfuric acid [15]| market for sulfuric acid | Cut-off, U

		refers to 98% H_2SO_4.Conversion calculations result in 412 kg of 98% H_2SO_4 in addition to water.	
Water	1944 kg	This is the water required to dilute 98% H_2SO_4. The estimated amount is based on theoretical stoichiometric calculations.	Tap water | market group for | Cut-off, U
Electricity (Leaching)	21.955 kWh	Electricity for leaching is retrieved from the EverBatt model documentation [17].The following parameters are used to estimate the electricity consumption of the leaching tank:<br>Design capacity = 0.10 ton/hour<br>Electrical power at design capacity = 2.983 kW<br>Feed = $m_{Alloy} + m_{H2O2} + m_{HCl}$	Electricity, medium voltage | market group for | Cut-off, U
Electricity (Filtration)	22.09	Electricity for filtration is retrieved from the EverBatt model documentation [17].<br>The following parameters are used to estimate the electricity consumption of the filter press:<br>Design capacity = 0.10 ton/hour<br>Electrical power at design capacity = 29.828 kW<br>Cu recovery rate from smelting = 99.8%<br>Fe recovery rate from smelting = 100%<br>(Retrieved from the single smelting step example in Umicore's patent [11])<br>Leaching efficiency = 95% (Assumed)<br>Total mass of copper = 72.08 kg<br>Total mass of iron = 6.02 kg (based on the GREET NMC111 BOM – This can vary depending on the battery feed composition, capacity, and used BOM)<br>$m_{filtered} = (72.08 \times 0.998 + 6.02 \times 1) \times 0.95 = 74.06$ kg	Electricity, medium voltage | market group for | Cut-off, U
**Co Solvent Extraction and Crystallization**			
Solvent (15% Cyanex272)	3112.97 L	Assuming a A:O ratio of 1 [21], the solvent volume should be equal to the aqueous solution volume. To calculate the volume of the incoming product solution, an average density of 1 kg/L is assumed.	
Solvent (15% Cyanex272) - Net	155.65 L	$V_{Solvent} \times (1 - f_{Regeneration})$<br>$f_{Regeneration} = 0.95$ [22]	
Electricity Crystallization	9.02 kWh	Calculated based on the rate 115.29 kWh/t Co [19]<br>1 ton of NMC111 LIBP contains 80.15 kg and a recovery efficiency of 98% is considered for Co [19].	Electricity, medium voltage | market group for | Cut-off, U

		⇨ m_{Co} = 80.15 × 0.996 × 0.98 = 78.23 kg	
Electricity (Reaction Workup)	146.99 MJ (40.83 kWh)	0.7 * $m_{product}$<br>$m_{product}$ =209.98 kg ($CoSO_4$)	Electricity, medium voltage | market group for | Cut-off, U
Steam (Reaction workup)	251.98 kg	1.2 x $m_{product}$<br>$m_{product}$ = 209.98 kg ($CoSO_4$)	Steam, in chemical industry | smelting and refining of nickel concentrate, 16% Ni | Cut-off, U
**Ni Solvent Extraction and Crystallization**			
Solvent (15% Cyanex272)	2,902.99 L	Assuming a A:O ratio of 1 [21], the solvent volume should be equal to the aqueous solution volume. To calculate the volume of the incoming product solution, an average density of 1 kg/m^3 is assumed.	
Solvent (15% Cyanex272) - Net	145.15 L	$V_{Solvent}$ × (1 - $f_{Regeneration}$)<br>$f_{Regeneration}$ = 0.95 [22]	
Electricity Crystallization	4.67 kWh	Calculated based on the rate 60.62 kWh/t Ni [19]	Electricity, medium voltage | market group for | Cut-off, U
Electricity (Reaction Workup)	144.98 MJ (40.27 kWh)	0.7 * $m_{product}$<br>$m_{product}$ =207.11 kg ($NiSO_4$)	Electricity, medium voltage | market group for | Cut-off, U
Steam (Reaction workup)	248.53 kg	1.2 x $m_{product}$<br>$m_{product}$ =207.11 kg ($NiSO_4$)	Steam, in chemical industry | smelting and refining of nickel concentrate, 16% Ni | Cut-off, U
Wastewater Treatment	0.422 m^3	-	Wastewater, average | market for wastewater, average | Cut-off, U
**Lithium recovery from flue dust (Refer the following sections for more information)**			
Distilled Water	1080 kg	We assume that water is added to have a liquid to solid ratio of 10 L/kg [14]<br>Assuming 70% of lithium is recovered in the flue dust => m_{Li2CO3}=108 kg	Refer to following sections
Carbon Dioxide (CO_2) (0.986 L/kg)	72.35 kg	The theoretical stoichiometric amount of CO_2 is calculated. An additional 10% is considered in the life cycle inventory estimation [14].	Carbon dioxide, liquid | market for | Cut-off, U
Electricity (Filtration)	32.18 kWh	Electricity for leaching is retrieved from the EverBatt model documentation [17].The following parameters are used to estimate the electricity consumption of the leaching tank:<br>Design capacity = 0.10 ton/hour<br>Electrical power at design capacity = 2.983 kW => $Electricity_{Filtration}$ = 0.298 kWh/kg * 108 kg	Electricity, medium voltage | market group for | Cut-off, U

Electricity/Heat for evaporation	-	Omitted due to the lack of data	-
CO_2 Emissions	65.77 kg	The theoretical stoichiometric amount of CO_2 is calculated.	Emissions to air

Life Cycle Inventory of Organic Solution (Inventory per liter of Organic Solution)

The inventory for solvent extraction is estimated based on a published study that looked into the separation of cobalt and nickel via solvent extraction using Cyanex272 [21]. The study consists of an experimental analysis to assess the variation of cobalt extraction efficiency using CYANEX 272 under different conditions. The authors vary the solution pH, solvent dilution, solvent saponification, and aqueous-to-organic solution ratio. According to this study, it is uncommon to use extractants in their pure form, and they are usually dissolved in an organic diluent. The table below summarizes the composition of the organic solution used, consisting of 15% Cyanex272, 5% modifier, and 80% Naphtha [21].

Table S17: Life cycle inventory of the organic solvent

Reference Input	Amount	Process used in SimaPro
Cyanex-272 (0.916 kg/L) (ml)	150	Refer the following section for Cyanex272 inventory
Modifier (Isodecanol) (ml)	50	Omitted
Naphtha (0.665 kg/L) (ml)	800	Naphtha | market for | Cut-off, U

Life Cycle Inventory of Cyanex272 Extractant (per kg of Cyanex 272) [25]

Table S18: Life cycle inventory of Cyanex272

Reference Input	Amount	Process used in SimaPro
3-methyl-1-butanol (kg)	1.9	3-methyl-1-butanol | market for | Cut-off, U
Sodium Hypophosphite (kg)	0.46	Refer to the following section
Electricity (kWh)	1.36	Electricity, medium voltage {US}| market group for | Cut-off, U
**Output**	**Amount**	
Cyanex272 (kg)	1	Reference Product
Calcium Carbonate (kg)	0.71	Emission to water

Phosphine (kg)	0.16	Emission to air

Life Cycle Inventory of Sodium Hypophosphite (per kg of Sodium Hypophosphite) [25]

Table S19: Life cycle inventory of sodium hypophosphite

Reference Input	Amount	Process used in SimaPro
Soda Ash (kg)	0.75	Soda ash, light, crystalline, heptahydrate | market for | Cut-off, U
Lime (kg)	0.52	Lime, hydrated, loose weight | market for lime, hydrated, loose weight | Cut-off, U
Phosphorus (kg)	0.56	Phosphorus, white, liquid | market for | Cut-off, U
Tap Water (kg)	0.25	Tap water | market group for | Cut-off, U
**Output**	**Amount**	
**Sodium Hypophosphite (kg)**	1	Reference Product

Life Cycle Inventory for Offgas Cleaning

The purpose of Offgas cleaning is to remove contaminants such as dioxins and furans. Most studies that assessed the pyrometallurgical recycling of Li-ion batteries, do not include the inventory for off-gas cleaning [25, 29, 33]. Multiple studies discuss the off-gas treatment methods. According to a critical review, offgas is treated differently cross the industry [34]. For example, in Umicore, the offgas is heated by a plasma torch, then it is sent to a post-combustion chamber where halogens are captured via injection of Calcium or Sodium based products. The offgas is then cooled down by water to avoid the recombination of organic compounds and the formation of dioxins and furans. On the other hand, in Inmetco, the authors argue that the offgas is scrubbed, and the scrubbing solution is sent to a wastewater treatment facility to recover heavy metals. Due to the lack of data, it is challenging to estimate an inventory for the aforementioned offgas treatment methods. One study assesses the life cycle environmental impact of Li-ion and Al-ion batteries and includes offgas treatment in the recycling process [35]. The authors assume that offgas cleaning occurs using activated carbon, and they develop an inventory that includes the activated carbon production, its recovery (re-activation), and the offgas cleaning step. The developed inventory is used in the current study and is summarized in the following table.

Table 20: Life cycle inventory of offgas cleaning

Reference Input	Amount	Process used in SimaPro
Crude coal (kg)	10.05	Hard coal {CN}| market for | Cut-off, U

Water (kg)	41.54	Tap water | market group for | Cut-off, U
Electricity (kWh)	1.84	Electricity, medium voltage | market group for | Cut-off, U
Steam (MJ)	44.56	Heat, district or industrial, natural gas | heat production, natural gas, at industrial furnace low-NOx >100kW | Cut-off, U
Hard coal ash (kg)	-0.6	Hard coal ash | treatment of hard coal ash, municipal incineration | Cut-off, U
**Output**	**Amount**	
Off-gas cleaning (pc)	1	Reference Process

Life Cycle Inventory of Lithium recovery from flue dust (per kg of Li_2CO_3 recovered)

Previous life cycle assessment studies do not consider lithium recovery in pyrometallurgical processes. However, Umicore has reportedly improved its UHT pyrometallurgical technology, and that includes improving its recovery efficiency and recovering lithium [36]. The latest Umicore patents do not describe the lithium recovery step [11, 12]. On the other hand, multiple publications have explored the lithium recovery from flue dust [37-40]. In one study, the authors develop a new method where the batteries are mechanically pretreated prior to smelting. In the smelting step, the battery feed is mixed with CaO and SiO_2 in an electric arc furnace (EAF) at a temperature of 1,750ºC. Lithium is reported in both, the slag (32%) and the flue dust (68%). Consequently, the slag is milled, mixed with the flue dust, and treated via acid leaching to recover lithium [40]. In another study, batteries are mechanically pretreated and copper slag is used a slag former in the smelting step. An electric arc furnace is used at a temperature of 1,450ºC, resulting in a 96.87% volatilization rate of lithium. The flue dust, containing LiCl, is collected and treated using water leaching to recover Li_2CO_3 [37]. In another study, the authors develop a pyrometallurgical recycling method and test it under laboratory and pilot scales. Pretreated spent batteries are mixed with dolomite, lime, and CaAl slag in an EAF at 1,600ºC. In the pilot-scale study, 68.3% of lithium is volatilized and recovered in the flue dust in the form of Li_2CO_3. Lithium carbonate is then leached using carbonated water.

The latter method is used in this study because it aligns the most with the Umicore process, in terms of material inputs into the smelting step and in terms of the Lithium recovery rate of 68% [39]. Also, the selected study recovers lithium only from the flue dust as Li_2CO_3. According to the Umicore patent, 100% of lithium is recovered in the slag [11]. However, lithium recovery from the slag is avoided in the industry because it is not economically feasible. Umicore claims that the updated pyrometallurgy process recovers more than 70% of lithium [36]. In this study, we will assume that 70% of lithium is recovered in the flue dust and the remaining 30% is lost in the slag.

The material and energy flows were first adopted from the pilot scale study, but that resulted in unrealistic results when applied to large scale applications. For instance, in the pilot scale study, the lithium recovery was tested for a small flue dust sample of 50 grams, containing approximately 35 g (68%) of Li_2CO_3. Alternatively, to fill this gap, the material and energy flows are based on a proxy process from a previous LCA study that assessed the reductive roasting recycling technology for Li-ion batteries [14]. The proxy process is a representative process for Li_2CO_3 recovery in this study. First, Li_2CO_3 has a limited water solubility, therefore, carbonated water leaching is used to convert it to $LiHCO_3$. The formed slurry is then

filtered, producing a filtrate and leaching residual. The filtrate, containing $LiHCO_3$, is then heated to release CO_2, evaporate H_2O, and to obtain the precipitated solid salt Li_2CO_3 following the equation below [14, 39]. The estimated inventory can be summarized in the table below.

$$2LiHCO_3(aq.) = Li_2CO_3(s) + H_2O(g) + CO_2(g)$$

Table S21: Life cycle inventory of carbonated water

Reference Input	Amount (Unit)	Process used in SimaPro
Distilled Water	10 (L)	Water, deionised {RoW| market for water, deionised | Cut-off, U
Carbon Dioxide (CO_2) (0.986 L/kg)	0.66 (kg)	Carbon dioxide, liquid| market for | Cut-off, U
Electricity (Filtration)	0.298 (kWh)	Electricity, medium voltage | market group for | Cut-off, U
**Output**		
**CO_2**	0.6 (kg)	Emissions to air

Carbon footprint, energy demand, water footprint, and toxicity associated with input materials/processes (Without electricity)

Table S22: Embodied environmental impacts of input materials

Chemical/Input	GWP (kg CO_2-eq/kg of product)[a]	Embodied Energy (MJ/kg of product)[b]	Water footprint – AWARE (m^3/kg of product)[c]	Water Footprint BEES+ (Liters/kg of product)	Freshwater toxicity (CTUe)	Abiotic Depletion Potential (kg Sb-eq)	*Electricity Input (kWh/kg)*[f]
**Oxygen Liquid (O_2)**	0.00509	80.6E-3	0.856	20.9	37.5	1.17E-7	**1.418 MV**
**H_2O_2**	1.05	17.4	3.08	72.6	1.26E4	1.44E-5	**0.56 MV**
**HCl**	0.486	10.9	0.714	20.7	6.56E3	1.86E-5	**0.209 MV**
**Nitrogen Liquid (N_2)**	0.00465	72.3E-3	0.354	8.3	20.1	5.62E-8	**0.563 MV**
**Carbon Dioxide Liquid (CO_2)**	0.523	4.46	0.111	2.97	4.18E3	1.28E-5	**0.4 MV**
**Solvent (15% Cyanex 272)[g]**	1.9E3	64.1E3	2.1E3	5.33E4	6.56E6	723E-5	**1.36 MV**
**Distilled Water**	0.00075	12.6E-3	0.041	1	3.82	3.16E-10	**0.091 MV**

* *The numbers used in the system dynamics model*

Carbon footprint, energy demand, water footprint, and toxicity associated with input materials/processes

Table S23: Embodied environmental impacts of input materials

Chemical/Input	Functional Unit	GWP (kg CO_2-eq/FU)	Embodied Energy (MJ/FU)	Water footprint – AWARE (m^3/FU)	Water Footprint BEES+ (Liters/FU)	Abiotic Depletion Potential (kg Sb-eq)	Ecotoxicity (CTUe)
**Offgas Treatment**	1 p	8.93	354	2.18	56.9	7.16E-6	1.56E5
**Limestone**	1 kg	0.00208	28.8E-3	0.00109	0.0265	8.61E-9	12
**Silica**	1 kg	0.0426	547E-3	0.00591	0.37	9.72E-8	157
**Steam**	1 kg	0.0127	175E-3	0.00255	0.0774	9.11E-8	1E3
**Tap Water**	1 kg	0.00075	12.6E-3	0.041	1	3.16E-10	3.82
**Wastewater Treatment**	1 m^3	0.543	6.68	-38.2	-895	6.48E-6	8.73E3

5. Additional Results: Environmental Impacts by Category

The charts below summarize the contribution by category to the environmental impact of battery recycling in North America. It's important to note that transportation is only included in conventional hydrometallurgy (refer to SI1 - section 1). Chemicals contribute the most to the environmental impact of recycling followed by electricity. When required, transportation is an important contributor, accounting up to 10% of the total environmental impact of hydrometallurgical recycling.

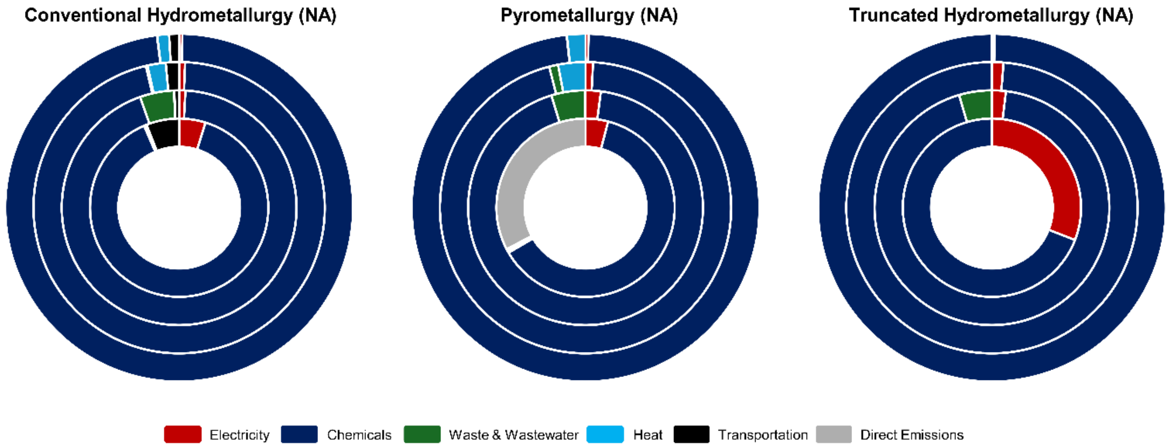

Figure S4: Contribution by category to the environmental impact of recycling in North America. Note: The chart rings from inside to outside represent: the carbon footprint, water consumption, freshwater toxicity, and abiotic depletion potential

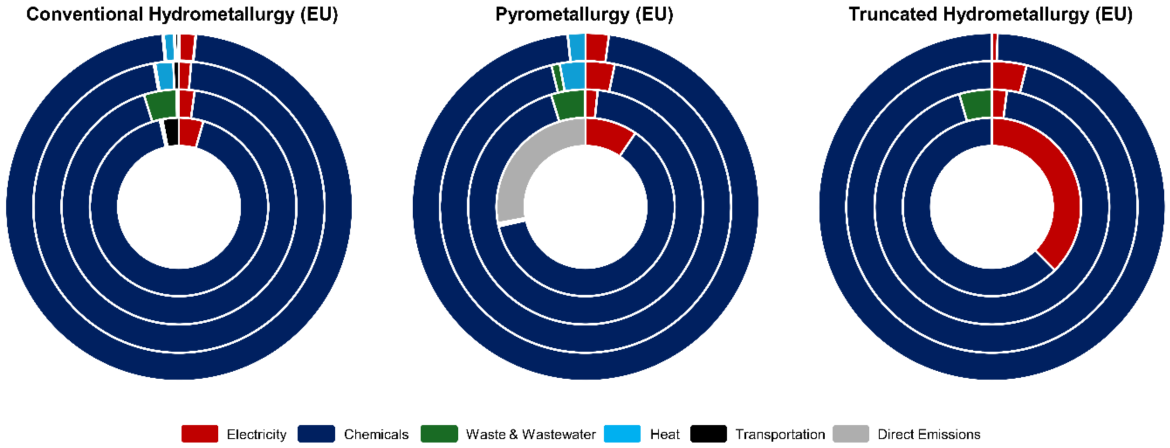

Figure S5: Contribution by category to the environmental impact of recycling in Europe. Note: The chart rings from inside to outside represent: the carbon footprint, water consumption, freshwater toxicity, and abiotic depletion potential

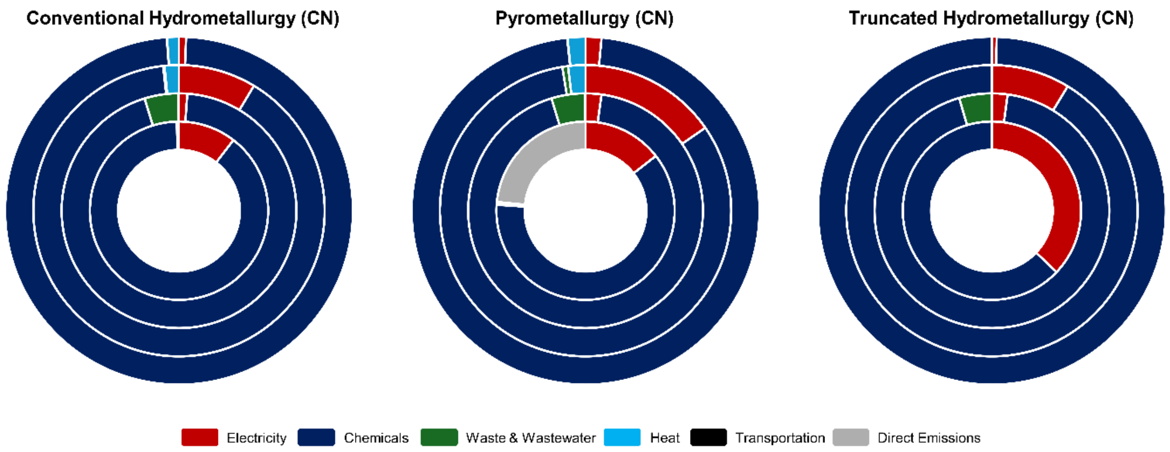

Figure S6: Contribution by category to the environmental impact of recycling in China. Note: The chart rings from inside to outside represent: the carbon footprint, water consumption, freshwater toxicity, and abiotic depletion potential

6. Literature Review

Table S24: Literature review summary

Title	Year	Reference	Battery Input	Recycling Process		Country/Geography	Functional Unit
				Hydro	Pyro		
**Examining different recycling processes for lithium-ion batteries**	2019	[41]	NMC622, NCA, LFP	x	x	US Average Grid, NWPP, RFCM	1 kg of recycled battery feed
**Comparative life cycle assessment of three recycling approaches for electric vehicle lithium ion battery after cascaded use**	2020	[42]	LMO, NMC, NCA	x	x	USA, NY	1 kWh of life cycle electricity delivered & 1 kWh of battery energy storage capacity
**Life cycle assessment of lithium-ion battery recycling using pyrometallurgical technologies**	2021	[28]	NMC111		DC Plasma Smelting	United Kingdom	Treatment of one ton of battery modules
					DC Plasma Smelting + Pretreatment		
					x		
**Comparative life cycle assessment of merging recycling methods for**	2021	[14]	Not specified		x	China	1 ton spent EoL LIBPs

**spent lithium ion batteries**				x			
**Simulation-based life cycle assessment for hydrometallurgical recycling of mixed LIB and NiMH waste**	2021	19	Mixed LIB + NiMH	x		Europe	1 kg of crushed waste batteries
**Impact of Recycling on Cradle-to-Gate Energy Consumption and Greenhouse Gas Emissions of Automotive Lithium-Ion Batteries**	2012	16	LMO		x		
**Life Cycle Assessment of an NMC Battery for Application to Electric Light-Duty Commercial Vehicles and Comparison with a Sodium-Nickel-Chloride Battery**	2021	43	NMC111, NMC622, and NMC811 (comparative analysis)		x	China	Two functional units are used: 1) 1 kWh of nominal energy capacity of the battery pack 2) 1 kg of battery pack<br><br>Results in next columns are based on FU#2
**Investigating carbon footprint and carbon reduction potential using a cradle-to-cradle LCA approach on lithium-ion batteries for electric vehicles in China**	2022	27	NMC811	x	x	China	1 kWh of the NCM811 battery for an EV

**Life cycle assessment of recycled NiCoMn ternary cathode materials prepared by hydrometallurgical technology for power batteries in China**	2022	44	NMC	x		China	Production of 1 kg NiCoMn ternary cathode materials
**Prospective LCA of the production and EoL recycling of a novel type of Li-ion battery for electric vehicles**	2019	45	Novel type of LIB - the Lithium Cobalt Phosphate	x		Europe	kWh of battery energy capacity
**Toward a cell-chemistry specific life cycle assessment of lithium-ion battery recycling processes**	2020	30	NMC111, LFP, NCA, SIB	x	x	Not specified	1 kWh of storag capacity
**Environmental Impact Assessment of LiNi1/3Mn1/3Co1/3O 2 Hydrometallurgical Cathode Recycling from Spent Lithium-Ion Batteries**	2022	46	NMC111	Hydrometallurgy - HCl	-	Average US grid mix was used	1 kg of treated cathode
				Hydrometallurgy - H_2SO_4/H_2O_2	-		
				Hydrometallurgy - H_3PO_4	-		

				Hydrometallurgy - Alkali Leaching $NH_3/(NH_4)_2SO_4/Na_2SO_3$	-		
				Hydrometallurgy - Alkali Leaching $(NH_4)_2SO_4$ for Mn	-		
				Hydrometallurgy - Organic Leaching $C_6H_8O_7/C_6H_{12}O_6$	-		
				Hydrometallurgy - Organic Leaching CH_2O_2/H_2O_2	-		
				Hydrometallurgy - Organic Leaching $C_3H_6O_3$	-		
				Hydrometallurgy - Bio-leaching	-		

**Life cycle assessment of recycling options for automotive Li-ion battery packs**	2022	29	NMC333	x	x	China	1 kWh of nominal battery pack capacity
**Comparative life cycle assessment of LFP and NCM batteries including the secondary use and different recycling technologies**	2022	33	LFP, NMC	x	x	China	1 kWh of nominal battery pack capacity
**Comparative life cycle analysis of critical materials recovery from spent Li-ion batteries**	2023	47	Not specified	x		Not specified	1 kg Li recovered from cathode powder of LIBs
**Environmental life cycle assessment of recycling technologies for ternary lithium-ion batteries**	2023	48	Not specified	x	x	China	1 kWh of ternary battery system
**Life Cycle Analysis of Battery Metal Recycling with Lithium Recovery from a Spent Lithium-ion battery**	2023	49	NCMxxx Specific chemistry not specified	x		Not specified	1 kg recovered LiOH<br>1 kg of produced NMC811 CAM
**Environmental assessment of secondary materials from battery recycling process chains: the influence of recycling**	2023	50	NMC111, NMC532, NMC622, NMC811	x		Europe	Two functional units are considered:<br>a- 1 kWh of battery produced<br>b- 1 kg of

**processe sand modelling choices**							battery pack produced
**Simulation-based life cycle assessment of secondary materials from recycling of lithium-ion batteries**	2023	51	Generic NMC is mentioned in the methodology. The authors report results mostly for NMC622 but they also discuss the results in comparison to NMC811	x		Europe	The production and recycling of one NMC-based battery 25 pack of 42.2 kWh to produce secondary battery-grade raw materials for direct re-use in battery 26 production.
**Co-products recovery does not necessarily mitigate environmental and economic tradeoffs in lithium-ion battery recycling**	2023	52	50% LCO, 20% LFP, 30% NMC	x		China	Recycling 1 kg of Co
**Life cycle assessment of hydrometallurgical recycling for cathode active materials**	2023	53	NMC111, LFP, LCO	x		Southeast USA. Grid modeled based on SERC	Two functional units are considered: a- 1 mAh of battery capacity produced

							b- 1 kg CAM recycled
**Combining dynamic material flow analysis and life cycle assessment to evaluate environmental benefits of recycling – A case study for direct and hydrometallurgical closed-loop recycling of electric vehicle battery systems**	2023	[20]	NMC111 and NMC811	x		Germany	1 kg spent traction battery
**Towards sustainable battery recycling: a carbon footprint comparison between pyrometallurgical and hydrometallurgical battery recycling flowsheets**	2023	[36]	NMC622	x	x	Average European Grid	Recycling of 100 kton of EOL LIB modules in Europe<br><br>The results are reported per kg EOL modules

References

1. Winjobi, O.; Dai, Q.; Kelly, J. C. *Update of Bill-of-Materials and Cathode Chemistry addition for Lithium-ion Batteries in GREET 2020*; Argonne National Laboratory: 2020.
2. NREL; NAATBatt, NAATBatt Lithium-Ion Battery Supply Chain Database. 2025, https://www.nrel.gov/transportation/li-ion-battery-supply-chain-database.html.
3. EEA. New registrations of electric vehicles in Europe 2024. https://www.eea.europa.eu/en/analysis/indicators/new-registrations-of-electric-vehicles (accessed 06/09/2025).
4. Taylor, B. Fortum to recycle EV batteries in Germany 2023. https://www.recyclingtoday.com/news/fortum-finland-germany-ev-battery-recycling-lithium-nickel-cobalt/ (accessed 06/09/2025).
5. Andritz, ANDRITZ and Duesenfeld sign cooperation agreement for lithium-ion battery recycling. Andritz: 2025. https://www.andritz.com/newsroom-en/recycling/2025-02-10-duesenfeld-group.
6. fortum, Fortum Battery Recycling opens Europe's largest closed-loop hydrometallurgical battery recycling facility in Finland. fortum: Finland, 2023. https://www.fortum.com/media/2023/04/fortum-battery-recycling-opens-europes-largest-closed-loop-hydrometallurgical-battery-recycling-facility-finland.
7. Ptak, A. US firm to build $1.3 billion battery materials plant in Poland 2025. https://notesfrompoland.com/2025/05/14/us-firm-to-build-1-3-billion-battery-materials-plant-in-poland/ (accessed 06/09/2025).
8. Miedreich, M., IONWAY – the Umicore/PowerCo JV – to locate its first production plant in Nysa, Poland. Umicore: 2023. https://www.umicore.com/en/media/newsroom/ionway-first-production-plant-in-nysa/.
9. Kochhar, A.; Johnston, T. G. Process, apparatus, and system for recovering materials form batteries. US 2020/0331003 A1. Oct. 22 , 2020, 2020.
10. Wang, Y.; Gratz, E.; Sa, Q.; Zheng, Z.; Heelan, J. Method and Apparatus for Recycling Lithium-Ion Batteries. US 10,522,884 B2. 2016.
11. Scheunis, L. Energy Efficient Pyrometallurgical Process for Treating Li-ion Batteries. 2022.
12. Scheunis, L.; Vermeulen, I. Pyrometallurgical process for recovering nickel, mananese, and cobalt. 2021.
13. Tran, T. T.; Moon, H. S.; Lee, M. S., Separation of Cobalt, Nickel, and Copper from Synthetic Metallic Alloy by Selective Dissolution with Acid Solutions Containing Oxidizing Agent. *Mineral Processing and Extractive Metallurgy Review* **2020,** *43* (3), 313-325.
14. Zhou, Z.; Lai, Y.; Peng, Q.; Li, J., Comparative Life Cycle Assessment of Merging Recycling Methods for Spent Lithium Ion Batteries. *Energies* **2021,** *14* (19), 6263.
15. Burrows, K.; Fthenakis, V., Glass needs for a growing photovoltaics industry. *Solar Energy Materials and Solar Cells* **2015,** *132*, 455-459.
16. Dunn, J. B.; Gaines, L.; Sullivan, J.; Wang, M. Q., Impact of recycling on cradle-to-gate energy consumption and greenhouse gas emissions of automotive lithium-ion batteries. *Environ Sci Technol* **2012,** *46* (22), 12704-10.
17. Dai, Q.; Spangenberger, J.; Ahmed, S.; Gaines, L.; Kelly, J. C.; Wang, M. *EverBatt: A Closed-loop Battery Recycling Cost and Environmental Impacts Model*; Argonne National Laboratory: 2019.
18. Li, Y.; Xie, S.; Zhao, Y.; Xia, L.; Li, H.; Song, S., The Life Cycle of Water Used in Flotation: a Review. *Mining, Metallurgy & Exploration* **2019,** *36* (2), 385-397.
19. Rinne, M.; Elomaa, H.; Porvali, A.; Lundström, M., Simulation-based life cycle assessment for hydrometallurgical recycling of mixed LIB and NiMH waste. *Resources, Conservation and Recycling* **2021,** *170*, 105586.

20. Rosenberg, S.; Kurz, L.; Huster, S.; Wehrstein, S.; Kiemel, S.; Schultmann, F.; Reichert, F.; Wörner, R.; Glöser-Chahoud, S., Combining dynamic material flow analysis and life cycle assessment to evaluate environmental benefits of recycling – A case study for direct and hydrometallurgical closed-loop recycling of electric vehicle battery systems. *Resources, Conservation and Recycling* **2023,** *198*, 107145.
21. Kihlblom, C. *Separation of cobalt and Nickel using CYANEX 272 for Solvent Extraction - In the presence of other contaminates in sulphate based leaching solution*; BOLIDEN, KTH: 2021; p 48.
22. Geisler, G.; Hofstetter, T. B.; Hungerbühler, K., Production of fine and speciality chemicals: procedure for the estimation of LCIs. *The International Journal of Life Cycle Assessment* **2004,** *9* (2), 101-113.
23. Morin, D.; Gagnebourque, C.; Nadeau, E.; Couture, B. Li-ion Batteries Recycling Process. 04 April 2019, 2019.
24. Gominšek, T.; Lubej, A.; Pohar, C., Continuous precipitation of calcium sulfate dihydrate from waste sulfuric acid and lime. *Journal of Chemical Technology & Biotechnology* **2005,** *80* (8), 939-947.
25. Wu, F.; Li, L.; Crandon, L.; Cao, Y.; Cheng, F.; Hicks, A.; Zeng, E. Y.; You, J., Environmental hotspots and greenhouse gas reduction potential for different lithium-ion battery recovery strategies. *Journal of Cleaner Production* **2022,** *339*, 130697.
26. ANL *GREET Model*, Argonne National Laboratory: 2023. https://greet.anl.gov/copyright.
27. Chen, Q.; Lai, X.; Gu, H.; Tang, X.; Gao, F.; Han, X.; Zheng, Y., Investigating carbon footprint and carbon reduction potential using a cradle-to-cradle LCA approach on lithium-ion batteries for electric vehicles in China. *Journal of Cleaner Production* **2022,** *369*, 133342.
28. Rajaeifar, M. A.; Raugei, M.; Steubing, B.; Hartwell, A.; Anderson, P. A.; Heidrich, O., Life cycle assessment of lithium-ion battery recycling using pyrometallurgical technologies. *Journal of Industrial Ecology* **2021,** *25* (6), 1560-1571.
29. Kallitsis, E.; Korre, A.; Kelsall, G. H., Life cycle assessment of recycling options for automotive Li-ion battery packs. *Journal of Cleaner Production* **2022,** *371*, 133636.
30. Mohr, M.; Peters, J. F.; Baumann, M.; Weil, M., Toward a cell-chemistry specific life cycle assessment of lithium-ion battery recycling processes. *Journal of Industrial Ecology* **2020,** *24* (6), 1310-1322.
31. Dunn, J. B.; Gaines, L.; Barnes, M.; Sullivan, J.; Wang, M. *Material and Energy Flows in the Materials Production, Assembly, and End-of-Life Stages of the Automotive Lithium-Ion Battery Life Cycle*; Center of Transportation Research, Argonne National Laboratory; Department of Mechanical Engineering, Pennsylvania State University: 2012.
32. Natarajan, S.; Aravindan, V., An Urgent Call to Spent LIB Recycling: Whys and Wherefores for Graphite Recovery. *Advanced Energy Materials* **2020,** *10* (37).
33. Quan, J.; Zhao, S.; Song, D.; Wang, T.; He, W.; Li, G., Comparative life cycle assessment of LFP and NCM batteries including the secondary use and different recycling technologies. *Science of The Total Environment* **2022,** *819*, 153105.
34. Pinegar, H.; Smith, Y. R., Recycling of End-of-Life Lithium Ion Batteries, Part I: Commercial Processes. *Journal of Sustainable Metallurgy* **2019,** *5* (3), 402-416.
35. Salgado Delgado, M. A.; Usai, L.; Pan, Q.; Hammer Stromman, A., Comparative Life Cycle Assessment of a Novel Al-Ion and a Li-Ion Battery for Stationary Applications. *Materials (Basel)* **2019,** *12* (19).
36. Van Hoof, G.; Robertz, B.; Verrecht, B., Towards Sustainable Battery Recycling: A Carbon Footprint Comparison between Pyrometallurgical and Hydrometallurgical Battery Recycling Flowsheets. *Metals* **2023,** *13* (12), 1915.
37. Qu, G.; Wei, Y.; Liu, C.; Yao, S.; Zhou, S.; Li, B., Efficient separation and recovery of lithium through volatilization in the recycling process of spent lithium-ion batteries. *Waste Manag* **2022,** *150*, 66-74.

38. Hu, X.; Mousa, E.; Tian, Y.; Ye, G., Recovery of Co, Ni, Mn, and Li from Li-ion batteries by smelting reduction - Part I: A laboratory-scale study. *Journal of Power Sources* **2021,** *483*.
39. Hu, X.; Mousa, E.; Ye, G., Recovery of Co, Ni, Mn, and Li from Li-ion batteries by smelting reduction - Part II: A pilot-scale demonstration. *Journal of Power Sources* **2021,** *483*.
40. Georgi-Maschler, T.; Friedrich, B.; Weyhe, R.; Heegn, H.; Rutz, M., Development of a recycling process for Li-ion batteries. *Journal of Power Sources* **2012,** *207*, 173-182.
41. Ciez, R. E.; Whitacre, J. F., Examining different recycling processes for lithium-ion batteries. *Nature Sustainability* **2019,** *2* (2), 148-156.
42. Tao, Y.; You, F., Comparative Life Cycle Assessment of three Recycling Approaches for Electric Vehicle Lithium-ion Battery after Cascaded Use. *Chemical Engineering Transactions* **2020,** *81*, 1123-1128.
43. Accardo, A.; Dotelli, G.; Musa, M. L.; Spessa, E., Life Cycle Assessment of an NMC Battery for Application to Electric Light-Duty Commercial Vehicles and Comparison with a Sodium-Nickel-Chloride Battery. *Applied Sciences* **2021,** *11* (3), 1160.
44. Du, S.; Gao, F.; Nie, Z.; Liu, Y.; Sun, B.; Gong, X., Life cycle assessment of recycled NiCoMn ternary cathode materials prepared by hydrometallurgical technology for power batteries in China. *Journal of Cleaner Production* **2022,** *340*, 130798.
45. Raugei, M.; Winfield, P., Prospective LCA of the production and EoL recycling of a novel type of Li-ion battery for electric vehicles. *Journal of Cleaner Production* **2019,** *213*, 926-932.
46. Iturrondobeitia, M.; Vallejo, C.; Berroci, M.; Akizu-Gardoki, O.; Minguez, R.; Lizundia, E., Environmental Impact Assessment of LiNi1/3Mn1/3Co1/3O2 Hydrometallurgical Cathode Recycling from Spent Lithium-Ion Batteries. *ACS Sustainable Chemistry & Engineering* **2022,** *10* (30), 9798-9810.
47. Mousavinezhad, S.; Kadivar, S.; Vahidi, E., Comparative life cycle analysis of critical materials recovery from spent Li-ion batteries. *Journal of Environmental Management* **2023,** *339*, 117887.
48. Tao, Y.; Wang, Z.; Wu, B.; Tang, Y.; Evans, S., Environmental life cycle assessment of recycling technologies for ternary lithium-ion batteries. *Journal of Cleaner Production* **2023,** *389*, 136008.
49. Yoo, E.; Lee, U.; Kelly, J. C.; Wang, M., Life-cycle analysis of battery metal recycling with lithium recovery from a spent lithium-ion battery. *Resources, Conservation and Recycling* **2023,** *196*, 107040.
50. Husmann, J.; Blömeke, S.; Cerdas, F.; Herrmann, C., Environmental assessment of secondary materials from battery recycling process chains: the influence of recycling processes and modelling choices. *Procedia CIRP* **2023,** *116*, 29-34.
51. Ali, A.-R.; Bartie, N.; Husmann, J.; Cerdas, F.; Schröder, D.; Herrmann, C., Simulation-based life cycle assessment of secondary materials from recycling of lithium-ion batteries. *Resources, Conservation and Recycling* **2024,** *202*, 107384.
52. Cao, Y.; Li, L.; Zhang, Y.; Liu, Z.; Wang, L.; Wu, F.; You, J., Co-products recovery does not necessarily mitigate environmental and economic tradeoffs in lithium-ion battery recycling. *Resources, Conservation and Recycling* **2023,** *188*, 106689.
53. Liu, Z.; Sederholm, J. G.; Lan, K.-W.; Cho, E. J.; Dipto, M. J.; Gurumukhi, Y.; Rabbi, K. F.; Hatzell, M. C.; Perry, N. H.; Miljkovic, N.; Braun, P. V.; Wang, P.; Li, Y., Life cycle assessment of hydrometallurgical recycling for cathode active materials. *Journal of Power Sources* **2023,** *580*, 233345.
